# Can Molecular Motors Drive Distance Measurements in Injured Neurons?

**DOI:** 10.1371/journal.pcbi.1000477

**Published:** 2009-08-21

**Authors:** Naaman Kam, Yitzhak Pilpel, Mike Fainzilber

**Affiliations:** 1 Department of Biological Chemistry, Weizmann Institute of Science, Rehovot, Israel; 2 Department of Molecular Genetics, Weizmann Institute of Science, Rehovot, Israel; ETH Zurich, Switzerland

## Abstract

Injury to nerve axons induces diverse responses in neuronal cell bodies, some of which are influenced by the distance from the site of injury. This suggests that neurons have the capacity to estimate the distance of the injury site from their cell body. Recent work has shown that the molecular motor dynein transports importin-mediated retrograde signaling complexes from axonal lesion sites to cell bodies, raising the question whether dynein-based mechanisms enable axonal distance estimations in injured neurons? We used computer simulations to examine mechanisms that may provide nerve cells with dynein-dependent distance assessment capabilities. A multiple-signals model was postulated based on the time delay between the arrival of two or more signals produced at the site of injury–a rapid signal carried by action potentials or similar mechanisms and slower signals carried by dynein. The time delay between the arrivals of these two types of signals should reflect the distance traversed, and simulations of this model show that it can indeed provide a basis for distance measurements in the context of nerve injuries. The analyses indicate that the suggested mechanism can allow nerve cells to discriminate between distances differing by 10% or more of their total axon length, and suggest that dynein-based retrograde signaling in neurons can be utilized for this purpose over different scales of nerves and organisms. Moreover, such a mechanism might also function in synapse to nucleus signaling in uninjured neurons. This could potentially allow a neuron to dynamically sense the relative lengths of its processes on an ongoing basis, enabling appropriate metabolic output from cell body to processes.

## Author Summary

Neurons have extremely long axonal processes that can reach lengths of up to 1 meter in human peripheral nerves. The neuronal cell body response to nerve injury is dependent on signals carried by molecular motors from the lesion site in the axon. The distance between the injury site and the cell body influences the type of response, suggesting that neurons must be able to estimate the distance of an axonal injury site, although how they do this is unknown. We have used a computational approach to model intracellular distance measurement after nerve injury. The models show the feasibility of a mechanism based on a rapid, near instantaneous, signal carried by action potentials in the nerve, followed by multiple slower signals carried on molecular motors. Such a mechanism can enable a neuron to discriminate between distances as close as 10% of total axon length. The model provides insights on retrograde injury signaling in neurons, including the biological relevance of the mechanism over different scales of nerves and organisms. Moreover, if similar mechanisms function in synapse to nucleus signaling in uninjured neurons, this could enable estimation of relative process lengths, thus guiding metabolic output from cell bodies to axons.

## Introduction

Neurons extend extremely long axonal processes that can exceed the diameter of the cell body by 4–5 orders of magnitude. This poses a unique challenge for intra-cellular signaling, since nerve cells require efficient transport mechanisms to move macromolecules and metabolites from the cell body to neurite terminals and back over distance. This communication problem becomes especially acute in the context of nerve injury, when the axon needs to provide the cell body with accurate and timely information regarding the site and extent of axonal damage [1]. Cell body responses to axonal injury are diverse, ranging from functional repair to cell death, and depend on both the intrinsic regeneration capacity of the neuron and responses to the local environment [2]–[4].

The distance of the lesion site from the cell body is one of the factors determining neuronal responses to injury. For some populations of neurons, a more proximal axotomy leads to greater regenerative response by the cell body ([5]–[7] and references cited therein). Lesion distance was also shown to influence specific molecular responses to injury, including activation of cell body kinases [8] and up-regulation of growth-associated genes [5], [9]–[12]. Interestingly, the precise effect of lesion distance on neuronal response may differ in diverse neuronal populations. For example, an optic nerve lesion study reported that the number of regenerating retinal ganglion cells is *inversely* correlated with distance of the lesion from the optic disc [13]. In long neurons from two species of fish, lesions close to the cell body induce death, while beyond a certain lesion distance neurons regenerate [14],[15]. Moreover, the lag time for initiation of regeneration in these neurons is tightly correlated with lesion distance [14],[15]. Taken together, these findings demonstrate that neurons from different functional classes and species have the capacity to differentiate between lesion sites at different locations in their axons.

Early workers in the field proposed a number of hypotheses to explain disparate cell body responses to differently located axonal lesions [16],[17]. Diffusion mediated signaling is not likely to function efficiently over the requisite distances [18], and other mechanisms like signaling waves [19] or spatial gradients of protein abundance [20] have not been demonstrated to occur over axonal distances. On the other hand, two long distance signaling mechanisms have been characterized in nerve injury paradigms- a rapid electrophysiological signal of short duration [21] and a second slower wave of signals transported on molecular motors [1],[22]. Motor-driven signaling has emerged as a versatile mechanism for long distance communication along nerve axons [23],[24], and in this study we have used computer simulations to examine the possibility that it can provide lesion distance information in injured neurons. The analyses support feasibility of a multiple signals model, wherein distance information is inferred from the time delay between the arrival of an electrophysiological fast signal and slow signals carried by the molecular motor dynein. The simulations indicate that this mechanism can enable nerve cells to distinguish between distances of 10% or more of their total axon length.

## Results

### Translating signal arrival to distance measurement

**Figure 1:**
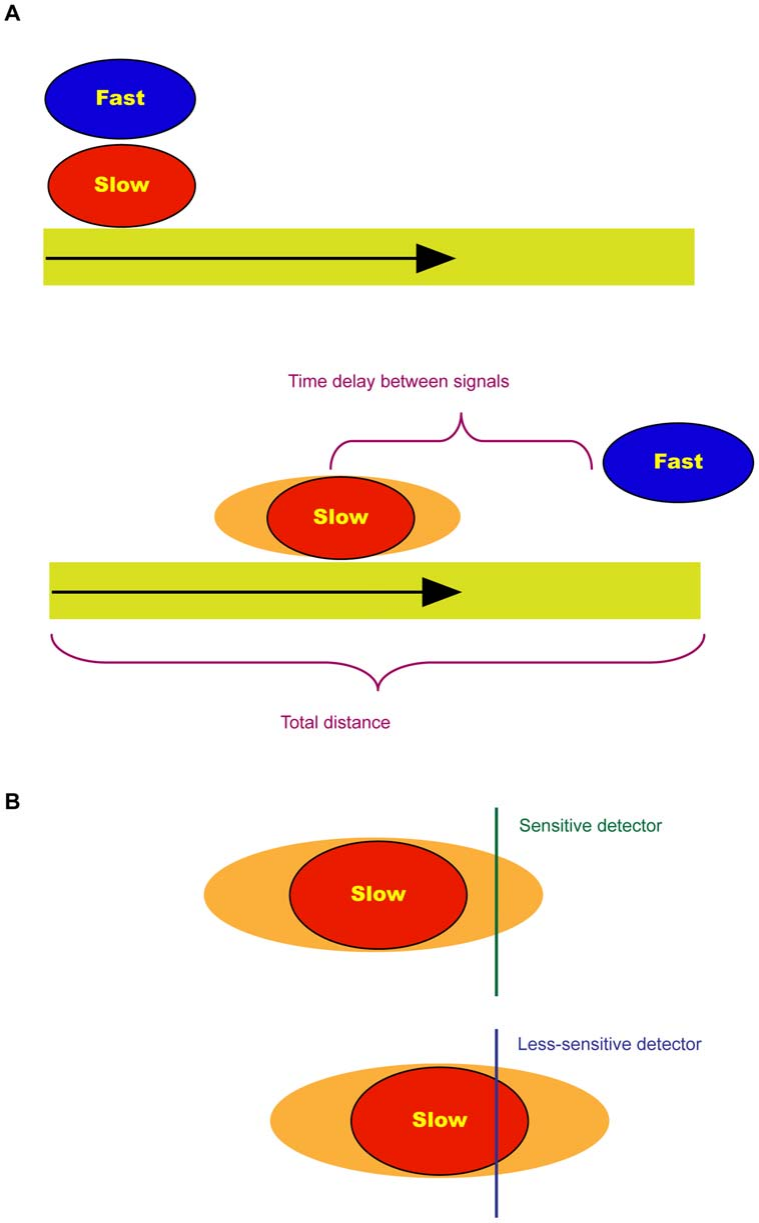
Possible mechanisms for assessing distance in injured neurons. (A) A two-signals model for distance assessment. This model assumes that an injury initiates two signals, fast and slow, which travel retrogradely from the site of injury. The time delay measured between the arrival of the fast signal and the arrival of a significant portion of the slow signal reflects the total distance traveled by the signals. Note that the slow signal is noisy, and therefore spreads over distance, hence does not arrive all at once. (B) A two-detectors model for assessing distances. This model assumes that a single slow signal is used to detect the distance from site of injury. The system utilizes two kinds of detectors: a sensitive detector responds to a small portion of the slow signal, and a less-sensitive detector requires a larger amount of the signal to arrive in order to initiate a response. The time delay between activation of the two detectors reflects the total distance traveled by the signal.

Inferring the distance traveled by a given signal can rely on two types of mechanisms, either quantifying chemical gradients over distance, or measuring the time delay between initiation of the signal and its arrival in the detection region. Although chemical gradients play central roles in biological systems, diffusion-based gradients cannot be established over axonal distances within a biologically relevant time frame after injury [25]. Thus, we examined mainly the second possibility, namely that a time delay between the initiation of a chemical signal in an injured axon and its arrival at the cell body can be interpreted by the cell as representing the distance traveled by the signal. In order for such a mechanism to work, it requires two reference points: an early time point representing the initiation of the signal, and a later time point representing the arrival of the signal. The latter requires a detection system that responds to the arrival of an amount of signal defined by a specified threshold, while for the former requirement, we hereby suggest two models that can in principle define signal initiation:

1. A multiple signals model, wherein the system measures the time delay between the arrival of a fast signal and the arrival of at least one additional slow signal (Figure 1A). The fast signal indicates occurrence of an injury, and thus initiates the time-delay measurement. Electrophysiological mechanisms are good candidates for rapid retrograde signals functioning on millisecond-second time scales after nerve injury [2],[26]. Any chemical signal will be orders of magnitude slower than an electrical signal, and thus for modeling purposes the fast signal can be regarded as noise-free (in model simulation, the entire fast signal arrives within a single time-step of the slow signal).
2. A multiple detectors model, wherein the system measures the time delay between the activation of at least two distinct detectors, which respond to the same slow signal (Figure 1B). According to this model, a sensitive detector responds to a small portion of the slow chemical signal, while a less sensitive detector will need to register the accumulation of a larger portion of the signal. The range of velocities and noise inherent in motor-driven signals [27] induce spreading of the signal over longer distances. This allows the system to derive distance information by using the initial fraction of arriving signal registered by the sensitive detector to provide a reference point for signal initiation, and the time of accumulation of most of the signal registered at the less sensitive detectors for estimating distance.

### Model parameters: sensitivity

Both models are based on measuring the arrival of a sufficient amount of the slow signal, defined as a fraction of 500 *in silico* particles moving in a Matlab-defined simulation environment (see methods). Since we do not have any data regarding the signal concentration required for initiating a response, our models explore a series of thresholds, defined as fractions of arriving signal from the total signal generated at the injury site. These sensitivity thresholds range between 1% and 90% of the injury signal (i.e., a 1% detector will respond to 1% of the originally generated signal, whereas accumulation of 90% of the signal is required for response of a 90% detector).

### Model parameters: dynein velocities

**Figure 2:**
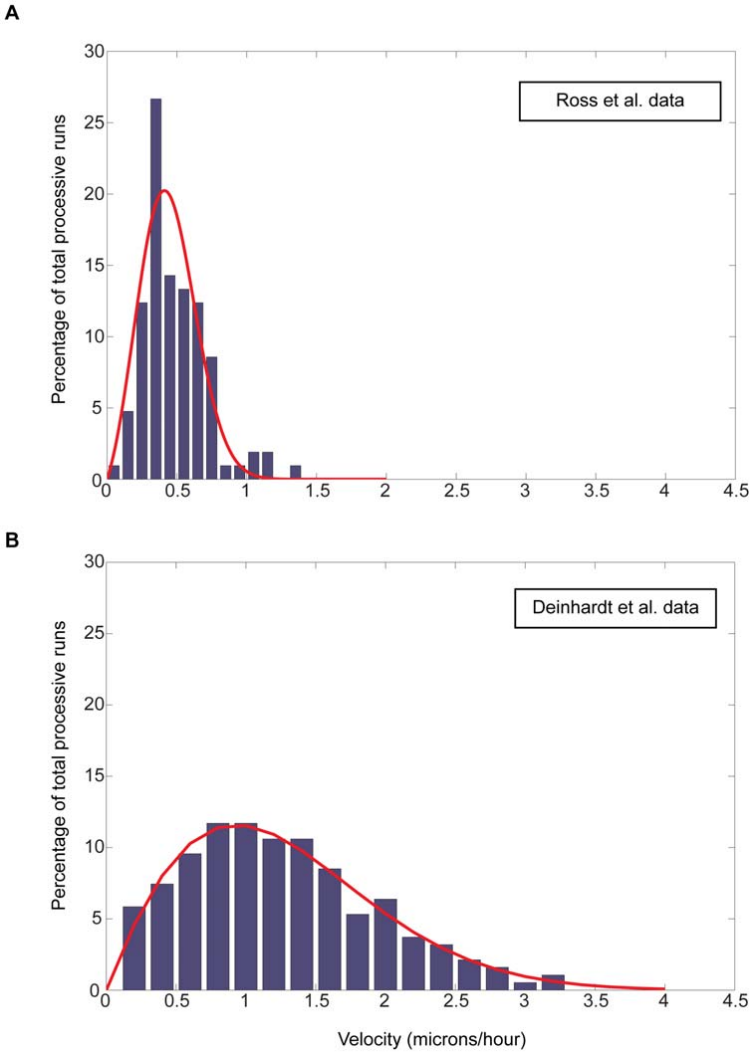
Distribution of dynein velocities. Experimental data (blue bars) were extracted from Ross et al. (A) and Deinhardt et al. (B). A curve fit procedure was applied to produce velocity distributions that were used in computer simulations (red lines).

Retrograde transport along the microtubule cytoskeleton in nerve axons is almost entirely dynein-based, thus the basic assumption in our models is that the slower signals are carried retrogradely as part of a dynein based complex. Dynein velocities have been measured in diverse systems from isolated molecules in vitro to in intact cells, and by different methods including direct imaging or end-point accumulation, leading to reports of a range of velocities from ∼0.5 µm/sec to ∼5 µm/sec [23],[27]. Our models require inputs of velocity distributions (rather than average velocities), and we therefore extracted velocity distributions from two experimental data-sets, one based on in vitro analyses of movement of individual dynein–dynactin–GFP complexes [28], and another that utilized cellular imaging of the retrograde transport of a GFP-labeled endosome marker in embryonic motor neurons [29]. For both data sets, a curve fit procedure was applied (see methods) resulting in a distribution function. Based on these distribution functions, random velocity values were assigned to migrating particles simulating dynein-trafficked retrograde signals. Figure 2 depicts the experimental data and the fitted distribution functions for both data sets. Unless otherwise specified, all simulations utilized the distribution function derived from the data of Ref. [28].

### Model construction

**Figure 3:**
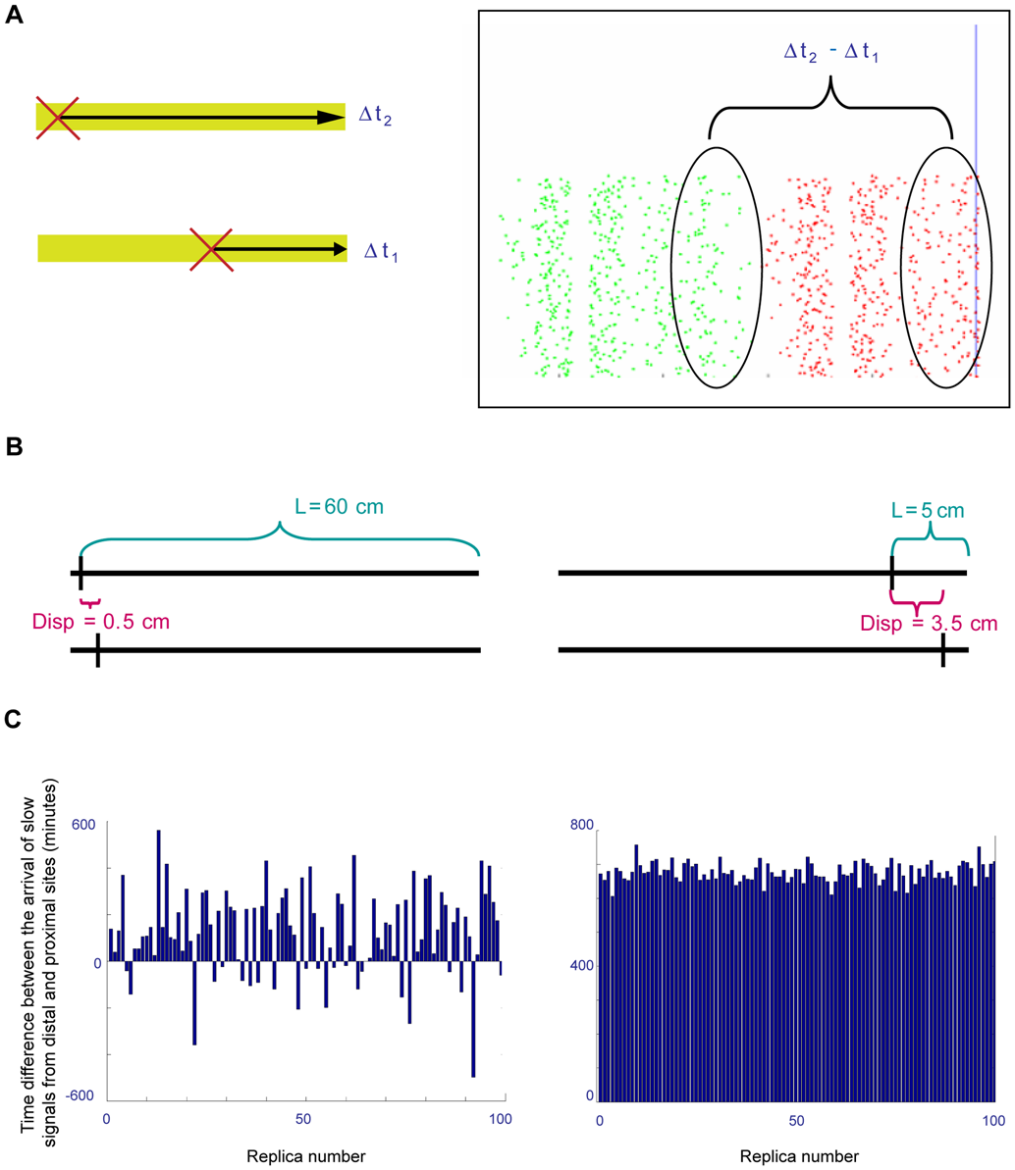
Modeling Approach. (A) Comparing distance measurements for distal and proximal injuries. Green and red dots represent the slow signal initiated by the distal and proximal injuries, respectively. These two sets of signal dots represent two distinct experiments (as shown on the left), and are shown together on the simulation screen due to technical considerations. (B) Model parameters for each simulation included total distance between the distal injury and cell body (L), and injury displacement – the distance between the proximal and distal injuries (Disp). Sites of injuries are indicated by vertical black bars. A case in which the displacement is small relative to the total distance (left) is assumed to be more susceptible to noise than a case in which the displacement is rather large in respect to the total distance (right). (C) Testing system sensitivity and reliability by simulation repetition. We repeated the simulations 100 times for each distinct set of conditions. Results are shown for the examples depicted in Panel (B).

As depicted in Figure 3A, our initial model system performs a comparative measurement. Two injuries are performed in two distinct cells in-silico. In one cell the injury is introduced in a proximal location, and in the other cell a distal injury is performed. In response to each of the two injuries, a slow signal emanates from the injury sites, propagating retrogradely towards the cell body. The system then measures the time delay between the fast and slow signals from the proximal location (Δt1) and the time delay between the fast and slow signals from the distal location (Δt2). The difference between Δt2 and Δt1 reflects the system's ability to distinguish between the two locations: the larger this difference, the better the system in terms of distance measurement.

The simulations explore the influence of two parameters on system performance: the distance between the two injuries (hereafter referred to as injury displacement), and the total distance between the distal injury and the detector (L, or total distance). Figure 3B depicts a schematic representation of two cases, one in which the displacement is small and the total distance is relatively large, and one in which the displacement is relatively large compared to the total distance. The intuitive prediction is that a biological system will find it more difficult to distinguish between the two injury sites in the former case rather than in the latter.

### Model performance: consistency

In order to assess consistency of model performance, we repeated each such in-silico experiment 100 times. In each such repetition, the same detector sensitivity, same total distance from cell body, and same injury-displacement distance were used. Differences between repetitions emerge solely from random assignment of dynein velocities to the slow signal particles. Figure 3C depicts two sets of such 100 repeats for the two-signals model (the same procedure was applied to the two-detectors model, data not shown). In the first case (Figure 3C, left panel), the parameters that were chosen were: L (total distance) = 60 cm, Disp (injury displacement) = 0.5 cm, and the detector-sensitivity threshold was set to 30%. Each vertical bar (dark blue) represents a single repetition of the simulation. The value obtained for each repetition represents the time difference Δt2−Δt1 in minutes. The negative bars observed for approximately one tenth of the repeats indicate that for these specific simulations the system infers mistakenly that the distal injury site is closer to the cell body than the proximal injury site. In another ∼10% of the repeats, the measured Δt2−Δt1 time difference in signal arrival is less than an hour. Since the different molecular events involved in both generating the retrograde injury signals at the site of injury and interpreting them at the cell body may take about an hour [30]–[32], such a time difference in signal arrival might be below the resolving power of an injured neuron (i.e., even though the signal from the proximal injury site traveled up to an hour less than the signal from the distal site, the accompanying events of signal production and/or processing may exceed this time difference, thus making it biologically irrelevant). Moreover, despite the fact that these are 100 repeats of the same injury and displacement distances, reproducibility of the measurement is clearly very poor. Thus, at least for this 0.5 cm displacement distance that is two orders of magnitude smaller than the 60 cm total injury distance, the initial model cannot discriminate between locations of the two injury sites. In the second case (Figure 3C, right panel), we set L to 5 cm and Disp to 3.5 cm, using the same sensitivity threshold of 30%. In this case, the system provided a consistent set of measurements, all ranging around 16–18 hours.

### Systematic analysis of distance-displacement combinations

Figure 3C depicts two extreme examples of model performance for two distinct combinations of total distance/injury displacement. In order to conduct a systematic exploration of model performance, we extended this analysis to cover a wide range of distance-displacement combinations. For each distance-displacement combination, we performed 100 simulation repeats as described above. From each such set of 100 simulations we discarded the worst 5%, and then chose the minimal Δt2−Δt1 time-difference value out of the remaining 95% of the repetitions (Figure S1, red circle). Note that in the examples of Figure 3C this minimum is a negative value for the left panel, while in the case of the right panel the minimum value is approximately 1000 minutes.

We then used the collection of minima points to plot a 3D graph in which the X and Y axes represent injury displacement and total distance, respectively, and the Z axis represents the minimal Δt2−Δt1 time difference value for each X–Y combination (Figure S1, lower panel). Such graphs can be used to answer two basic questions regarding the models- first, can a given model distinguish between two distinct injury locations. This is determined by setting a cutoff for system failure due to either mistaken identification of the distal injury site as being closer than the proximal (resulting in a negative Z axis value), or a time delay that is too small to enable a differential biological response. Since differential biological responses to injury typically require transcription and translation, for purposes of the modeling the system cutoff was defined as a time delay of at least 60 minutes.

The second issue addressed by the 3D plots is whether a given model is consistent, i.e. will it provide a similar assessment for the same injury displacement, regardless of its distance from the cell body? This is reflected in the smoothness of the graph. In an ideal system, the time difference in the arrival of a signal that travels a distance x and a signal that travels a distance x+Δx should remain constant, regardless of the value of x. Thus for an ‘ideal’ 3D graph (Figure S2), straight lines along the X axis indicate consistency (i.e., for a given value of injury displacement, the time difference (Z) should remain the same at all total distance values). In order to assess the smoothness of a 3D graph plotted from the simulations, we use a root mean square deviation (RMSD) measurement. Given two sets of *n* points *v* and *w*, the RMSD is defined as follows:
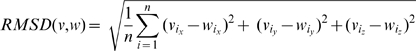
When calculating the RMSD for a model-generated graph compared to an ideally smooth graph, the lower the obtained RMSD value, the closer the graph to the ideal, hence the effects of changing parameters and models can be inferred from their comparative RMSD values.

### Initial models: two signals versus two detectors

**Figure 4:**
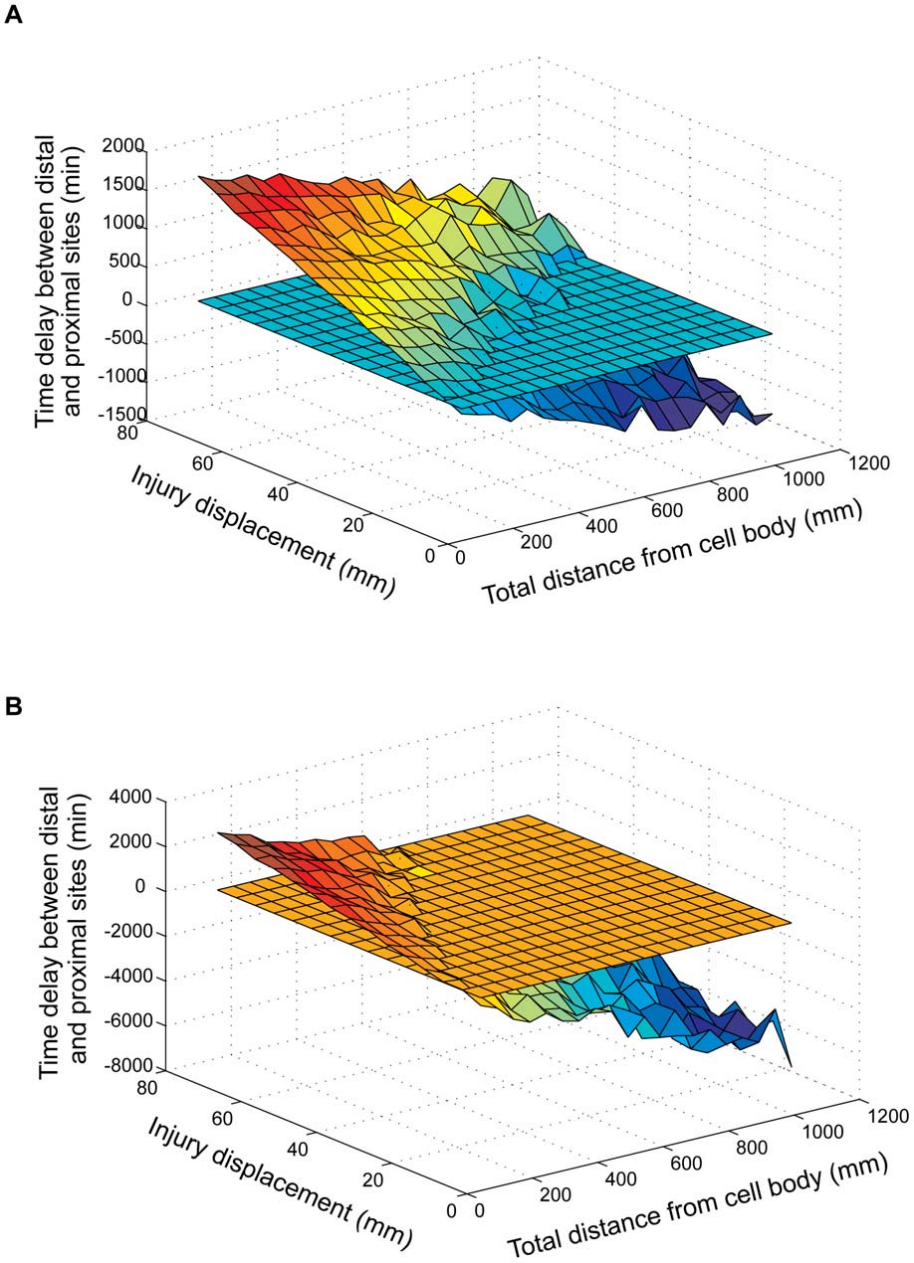
Best performance of the two signals versus two detectors models. (A) Best performance of a two-signals model (detector sensitivity set at 10%) The horizontal plane represents a 60 minute cutoff, below which the time difference measured by the system is not likely to enable a differential biological response. (B) Best performance of a two-detectors model, obtained for a system configuration of two detectors with 5% sensitivity threshold and 80% sensitivity threshold. Performance graphs for additional sensitivity permutations of these two models are shown in Figures S3 and S4, respectively.

Systematic exploration of the two-signals model showed that although it can function over part of the total distance/injury displacement combinations, the system failed over a significant portion of the distances range tested (Figure 4A, Figure S3). Furthermore, for a given injury displacement, the time-delay measurements did not show consistency over increasing total-distance values. For example, the ability of the system to detect an injury displacement of 8 cm decays with distance along the axon, and is essentially lost at total distances of 70–80 cm and above. RMSD values for a wide range of detector sensitivity thresholds indicate that the system performed better at sensitivity settings of up to 30%, and worsened significantly in the range from 40% to 80% (Figure S3).

Performance of the two-detectors model was much poorer, and in the best case the system detected injury location differences for only approximately one third of total distance/injury displacement values (Figure 4B, Figure S4). Unfortunately, the RMSD measurement seems to be uninformative for comparing different permutations of the two-detectors model. Rather than reflecting model performance, RMSD values reflect the ‘gap’ between the sensitive detector and the insensitive detector. The larger the difference between the thresholds of the two detectors, the larger the time difference between the distal and proximal locations. Thus, two 3D graphs that are similar in terms of smoothness, but differ in their Z values (time differences) will yield different RMSD values (Figure S4).

### Model improvement: integrating multiple slow signals

**Figure 5:**
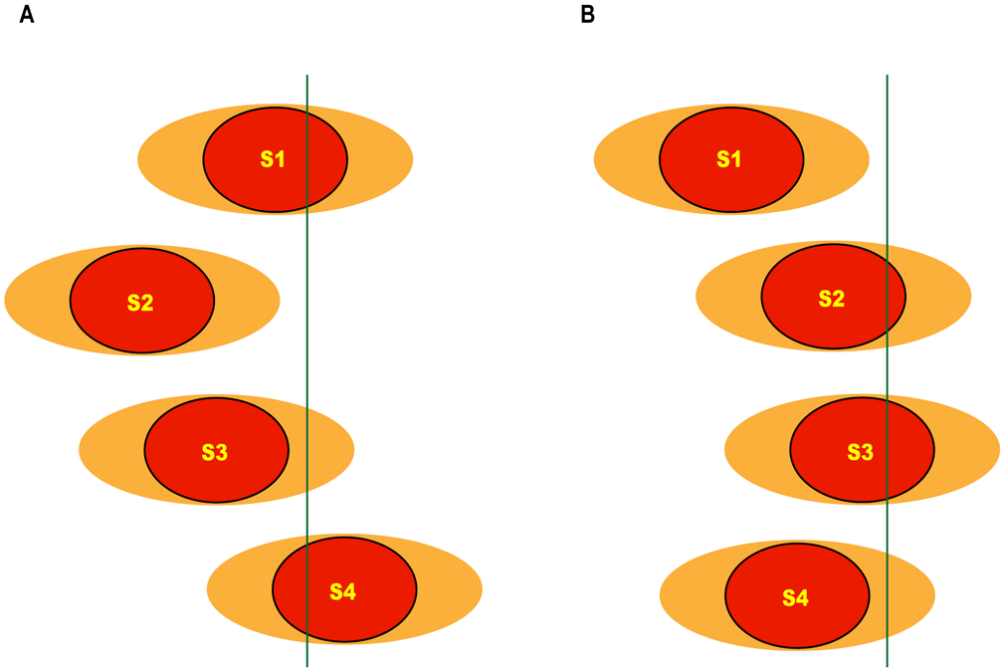
A multiple slow-signals model. This model assumes that several slow signals travel retrogradely from the site of injury towards the cell body. In order to initiate a response, a significant portion from a subset of these signals needs to arrive at the detection area. The figure depicts two examples of a situation in which a significant portion of two out of four signals has already arrived: (A) S1 and S4, and (B) S2 and S3.

**Figure 6:**
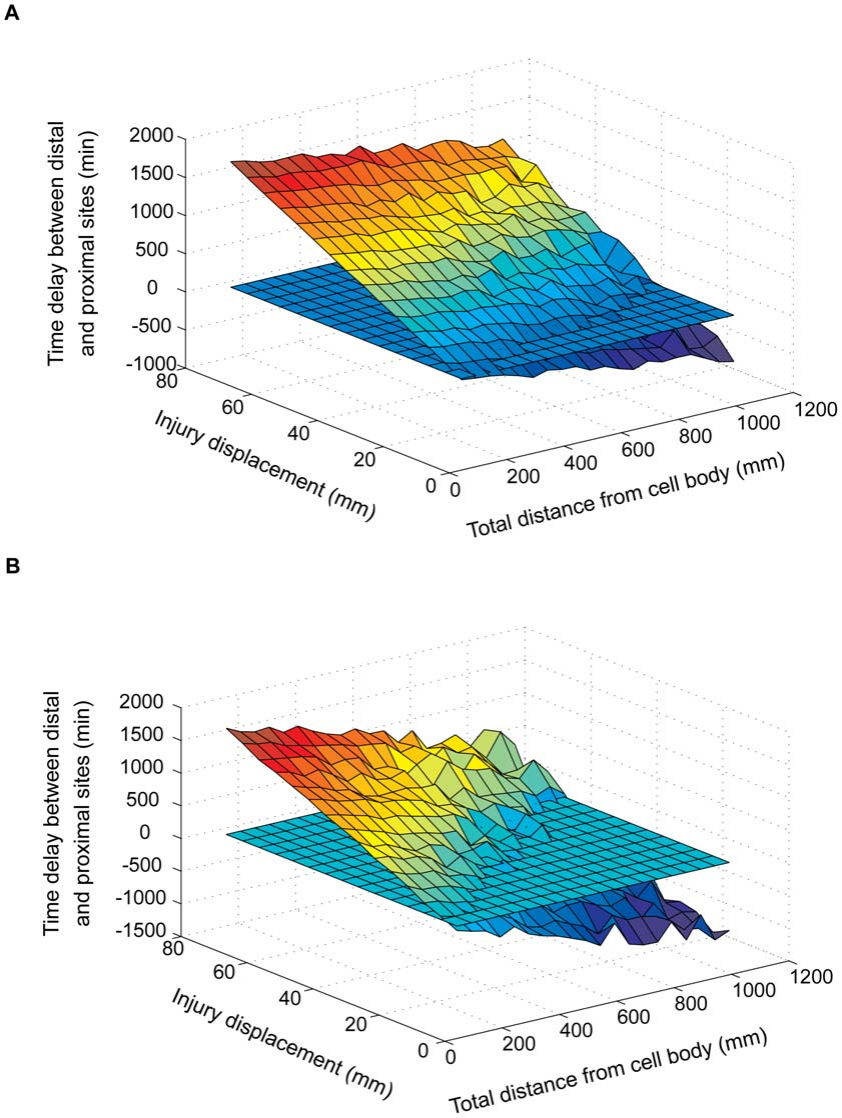
Improved performance of a multiple slow signals model. Integrating three out of six slow signals (A), resulted in a smoother graph than in a single slow-signal simulation (B), with RMSD values of 1220 versus 356, respectively. Moreover, the number of successful measurements was also higher, as reflected by the fraction of the graph above the horizontal 60 minutes cut-off plane. Both graphs correspond to a 10% detector sensitivity threshold. Performance graphs for additional sensitivity permutations of the multiple slow signals model are shown in Figure S5.

Since model performance in two signals or two detectors mode was not satisfactory, we modified the two-signals model to include several slow signals rather than a single slow signal, and assume that an effective response is triggered when a subset of these signals arrives at the cell body (Figure 5). From a biological point of view, this may reflect a situation in which there are several dynein-carried signals. We further assume that as far as detector-sensitivity is concerned, there is no significant difference between the signals (i.e., in terms of our model they utilize similar detection systems). The rationale behind this modification is that in a noisy system, multiple measurements are expected to be more accurate than a single measurement. In its original configuration, in order for a distal injury to be identified by the system as a proximal one, it was sufficient that a small fraction of the slow signal particles emanating from the distal site would randomly acquire higher velocities than the signal particles originating from the proximal point. In order for a similar phenomenon to occur in the multiple signals system, the distal point needs to randomly “win” not only once, but in several slow-signal velocity acquisitions. Figure 6 compares the performance of a system with six slow signals, of which any three will initiate a response, versus performance of the previously described system with a single slow signal. A significant improvement is observed in consistency (graph smoothness), together with a marked increase in the total distance and injury displacement ranges for which the system attains a successful outcome (Figure 6A and Figure S5). RMSD values are also significantly improved (Figure S5).

We considered examining a similar extension of the two-detectors model to multiple detectors. However, whereas extending the two-signals model to multiple signals did not require any new (and unjustified) assumptions regarding system parameters, a similar extension of the two-detectors model requires overly speculative assumptions. Consider, for example, a system with three kinds of detectors with sensitivity thresholds s1, s2, and s3, where s1<s2<s3 (i.e., s1 is the most sensitive detector). The limiting determinant of system performance will have to be the time delay between activation of s1 and s3 – having s2 as an intermediate detector will not influence the result, unless one assumes preferential effects of such intermediate detectors. In the absence of any data, such speculative configurations may be completely detached from biological reality. Nonetheless, we did try to modify some quantitative features of the slow signal, in order to check whether the poor performance of the two-detectors model results from the specific biological datasets that were used in this work. We applied the following modifications to the model: (i) using a uniform distribution of velocities instead of the data-based Gaussian distributions, (ii) using velocities 1–3 orders of magnitude faster than the data-based velocities, and (iii) using wider and narrower velocity distributions (obtained by modifying the parameters of the curve-fit functions described in the Methods section below). None of these modifications yielded any significant improvement in model performance (data not shown). It therefore seems that the the two (or multiple) signals model is qualitatively superior to the two-detectors model, and the difference in model performances cannot be attributed to a quirk of specific model configuration.

### The effects of changing data-sets on model performance

**Figure 7:**
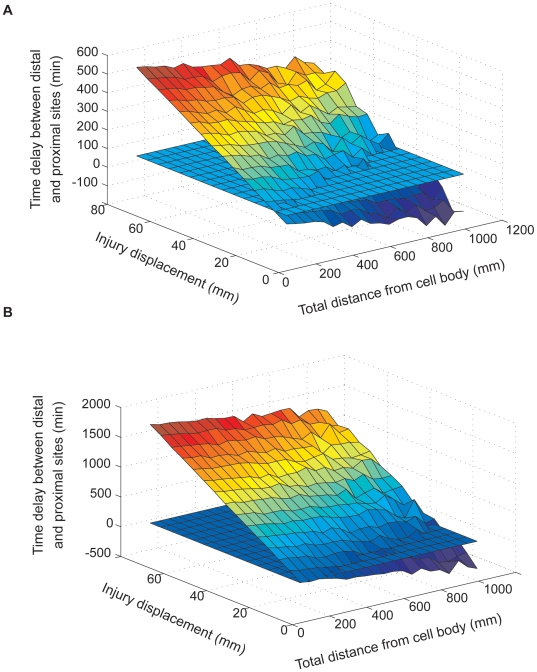
Comparing two dynein velocity data sets. The figure depicts the results of executing a model with a 10% sensitivity threshold detector, based on the Schiavo lab dataset (A) and the Holzbaur lab dataset (B). Executions were performed for a model configuration that integrates 5 out of 10 slow signals.

As noted above, we used two sets of dynein velocity measurements for our modeling work: a data-set from Ross et al. [28], representing velocities of isolated dynein-dynactin complexes in vitro, and a data set from Deinhardt et al. [29], based on tracking of GFP-labeled tetanus toxin in live motor neurons. The average dynein velocity measured by Deinhardt et al. was higher than the average dynein velocity measured by Ross et al. – 1.3 µm/sec and 0.45 µm/sec, respectively, and the velocity distributions of Deinhardt et al. spanned a broader range (Figure 2). As a consequence, time delays between the arrival of signals from distal and proximal locations in simulations based on the Deinhardt et al. data were smaller than in simulations based on the Ross et al. data, and simulations based on the Deinhardt et al. data were more susceptible to noise (Figure 7). Thus, in a system configuration integrating five out of ten signals (a model configuration based on multiple slow signals – see also Figure 5 and accompanying text above), simulations based on the Ross et al. data yield satisfactory results over a broader combination of distances and injury displacements than simulations based on Deinhardt et al. 's data (Figure 7). Nonetheless, increasing the number of signals and detector sensitivities for the Deinhardt et al. data show the same trends for improvement as demonstrated for simulations based on Ross et al. (data not shown). Thus, it is reasonable to assume that optimal results can be obtained also from relatively noisy motor behavior given a sufficient number of signals and appropriate detector sensitivity.

## Discussion

The distance between the site of injury and the cell body seems to have a significant effect on a neuron's ability to recover from mechanical injury [14]–[17],[33],[34]. Furthermore, there are both qualitative and quantitative aspects to this distance effect. In specific neuron types, once distance between cell body and site of injury drops below a certain lower threshold, no regeneration occurs, whereas above this threshold the probability of regeneration increases continuously with the increase in distance between the cell body and site of injury [14],[15]. In other neuronal populations, a more proximal axotomy leads to greater regenerative response by the cell body [5]–[7]. Despite the clear biological significance of injury distance in neural tissues, the mechanism by which distance from the site of injury is measured is unknown, and the degree of precision required from such a measurement is not clear.

In this work, we aimed at providing a theoretical framework for examining how intracellular distance measurement might be accomplished at the cellular level within a neuron. Computer simulations based on existing biological data were used to examine these concepts, and to assess their plausibility. Nonetheless, we are fully aware that the results and conclusions presented in this paper were derived from models that are abstractions of the real biological system, although we tried to keep speculations regarding the the mechanisms driving the behavior of these models to the bare minimum. We should also note some of the limitations of our approach, thus for example dynein velocity might be influenced by the type of cargo [35]. Although this was not factored into our models, the analyses show that the differences between the two velocity distributions used for model simulations do not affect key qualitative behaviors of the system (Fig. 7). Another issue not explicitly modeled is processivity of the dynein motor, namely the propensity of the motor to stall, or to move over limited distances in the opposite direction [28],[36]. In the above described simulations, signaling molecules were assigned a given velocity, and they continued moving retrogradely with that velocity throughout the entire simulation. We carried out initial tests of the effects of motor pausing behaviors by running simulations at which in each time step 30% of the particles were randomly selected to remain in the same position until the next time step (Figure S6). This modification did not seem to have any significant effect on model performance. We further examined the effect of switching velocities in the model by re-assigning velocities to 10% of the molecules once per 100 time steps (a typical simulation is of the order of 10^4^ time steps). As can be seen in Figure S7, this modification improved the performance of the system in terms of failure percentage. This can easily be understood by considering that if a given signaling molecule undergoes velocity switches for sufficient time, eventually the velocity of each molecule will converge to the average velocity of the entire population, decreasing noise in the system. Thus, our main findings without considering the possibility of velocity switching may actually reflect a worst-case scenario.

Despite the above caveats, the modeling shows that in principle a set of dynein-mediated signals can provide intracellular distance information in an injured neuron. Furthermore, we did not have to add any “external players” to or impose speculative mechanisms on the model. Both the fast electrical signal and the slow chemical signal have been characterized in the context of nerve cell injury [21]. Moreover, such a mechanism might also function in synapse to nucleus signaling in uninjured neurons if a neurotransmitter or other synaptic stimulation elicits electrical (fast) signals concomitantly with dynein-based (slow) signals. Such a scenario has actually been reported for the neurotrophin BDNF, which elicits both rapid electrophysiological signals [37] and dynein-transported signaling endosomes [38]. NMDA receptor signaling provides another example, transmitting both acute electrophysiological signals [39] and activating macromolecule transport by importins and dynein [40],[41]. If such signaling systems are indeed used to sense synapse to nucleus distance, this would allow autonomic length measurements of neuronal processes on an ongoing basis, which in turn could guide metabolic output from neuronal cell bodies to processes.

**Figure 8:**
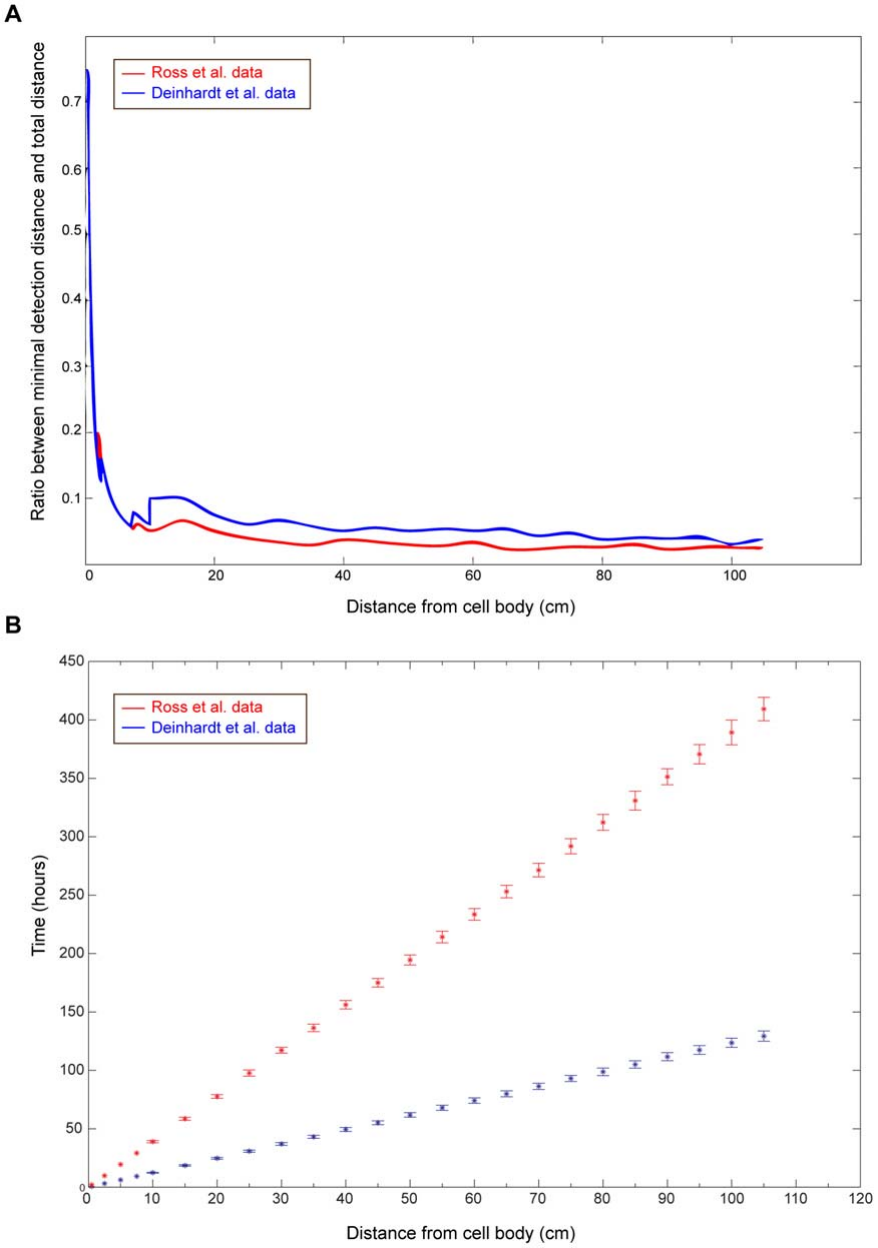
Minimal displacement detection as a function of total distance. In order to assess the best resolution at which distances can be detected for various axon lengths, we refer to the biological cutoff measurement described above. For each axon length, the corresponding minimal displacement value for which the time difference was above the cutoff was calculated. Results are presented in terms of the percentage of a given axon length that can be detected (rather than absolute distance values). Starting from an axonal length of ∼4 cm, the system can detect displacements which are 5–10% of the total axon length. Results are shown for measurements based both on the Ross et al. data (blue) and on the Deinhardt lab data (red).

The existence of cellular mechanisms that detect time delays between signaling events has been shown to exist in diverse biological systems (e.g. [42],[43]). Even the expansion of the model to multiple slow signals reflects the existence of multiple signaling complexes which are retrogradely transported by dynein [1],[22],[24]. The proposed model can fit a large range of nerve lengths, covering a diversity of organism sizes. Finally, the models allow two firm conclusions that might be testable experimentally in the future; first, that use of multiple and partly redundant signaling entities provides a more robust distance assessment mechanism measurement than a single signal, and second that distance detection resolution is proportional to neurite length (Figure 8). It will be intriguing to follow experimental testing of these ideas in the future.

## Methods

### Data fit

In order to produce a velocity distribution function, a data fit procedure was applied to the two experimental datasets used in this work. Both datasets were obtained from analyses of the movement of individual molecular complexes – either in vitro [28] or in live neurons [29]. In both cases, the authors reported their results as the relative occurrence of given ranges of velocities (e.g., 5% of the observations were at velocity ranges of 0–0.2 microns/sec) over a non-exhaustive number of molecular complexes (148 discrete complexes in Reference [28], 126 in Reference [29]). Thus, the reported velocity sets are not an ideal representation of velocity distribution, but rather an experimentally limited sampling. The curve fit procedure allowed us to compute a continuous function which could then be used to randomly assign velocities to the signaling molecules in each simulation round. For this purpose, we used a built-in Matlab script (*fminsearch*) based on the Nelder-Mead method [44],[45]. Since our model does not acount for zero velocities, we introduced a slight modification to the Gaussian function, thus requiring the velocity distribution function to intersect with (0,0). The curve fit function that was used was of the form:
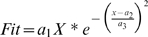
Thus, for the velocity X = 0, the function yields zero occurrences.

Goodness-of-fit was assessed by calculating the root mean square deviation (RMSD) between the observed data points and the values predicted by the calculated function:
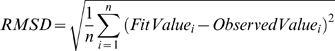
where the values are given in terms of percentage-of-occurrence of given dynein velocities (see Fig. 2). This measurement provides an estimate for the average distance between a given data point and the calculated curve.

### Fit results

For the Ross et al. data set, the following results were obtained:
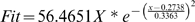
RMSD = 3.1%

For the Deinhardt et al. data the following results were obtained:
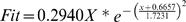
RMSD = 0.61%

### Model construction

Molecular transport complexes are represented as moving particles. Each such particle has a location in space, and it can move according to its velocity. This approach also allows extension of the model in the future to include additional molecular properties and experimental data. In our model, a signal is composed of 500 moving particles. In order for a signal to achieve its effect, a minimal fraction of the signal should arrive at the detector, this is presented as detector sensitivity in % in the results section. The influence of various sensitivity thresholds was examined during simulations.

### Simulation runs

All simulation scripts were written in MATLAB, and simulation executions were performed on the Wiccopt cluster (hosted by The Weizmann Institute's computing center) to allow parallel executions of simulations which varied in initial parameter settings. The Cluster's nodes consist of machines with: 2 quadcore xeon CPU's, 1 quadcore xeon CPU, 2 dualcore AMD opteron, and 1 dualcore AMD opteron.

## Supporting Information

Figure S1 Systematic analysis of total distance and injury displacement combinations. The X axis represents total distance from cell body (mm), the Y axis represents injury displacement (mm), and the Z axis represents the minimal (i.e. worst case) Δt2−Δt1 time-difference value (in minutes) out of 100 simulation repeats obtained for each X–Y combination. The figure shows results for a 20% sensitivity threshold in a multiple-signal based model.
(0.34 MB PDF)

Figure S2 An “ideal” graph. Each data point represents the mean Δt2−Δt1 time-difference value (in minutes) of 100 simulation repeats obtained for each X–Y combination. The figure shows data for a 20% sensitivity threshold in a multiple-signal based model.
(0.15 MB PDF)

Figure S3 The influence of detector sensitivity threshold on a two-signals model performance. Simulations were run for a wide range of detector sensitivity thresholds, revealing an optimal performance at the range of 10%–20%. RMSD values are depicted for each model configuration.
(1.27 MB PDF)

Figure S4 The influence of detector sensitivity threshold on a two-detectors model performance. A wide range of detector sensitivity combinations was examined, but failed to exceed a success rate of 35% in distinguishing between proximal and distal injuries. (A) Depicted examples include the following detector sensitivity threshold combinations: 5% and 60%, 5% and 80%, 10% and 70%, and 10% and 60%. (B) Failure percentage in various combinations of two detectors. The X axis (left) represents the sensitivity threshold of the more sensitive detector (Detector 1), whereas the Y axis represents the sensitivity threshold of the less sensitive detector (Detector 2). The lowest failure percentage was received for the 5%-and-80% configuration. Configurations in which the two detectors had relatively similar sensitivity threshold (back diagonal) gave the poorest performance.
(1.51 MB PDF)

Figure S5 Influence of detector sensitivity on performance of the multiple signals model. Simulations were run for a wide range of detector sensitivity thresholds, revealing an optimal performance in the range of 10%–40%. RMSD values are depicted for each model configuration. Note that not only did model extension improve the performance of a given detector sensitivity threshold, but moreover the worst performing configuration of the multiple signals system was still better than the best performance of the single slow signal system. In addition, the range of “optimal detectors” in a multiple-signals system is wider - 10–40% compared to 10–20% for the original system (see Fig. S3).
(1.44 MB PDF)

Figure S6 Evaluation of the effect of dynein pauses in the two-signals model. The velocity distributions depicted in most of our analyses refer only to positive dynein velocities, although it has been shown that dynein movement may also include pauses (velocity = 0), as well as limited movements in the opposite direction (i.e., negative velocity). We therefore ran a set of simulations in which 30% of the particles were randomly assigned to pause at any given time step. Paused particles resumed movement at their originally assigned velocity at the subsequent time step. Panels A and B depict the results of simulations for a model configuration with one slow signal and a detector sensitivity of 20%, without pauses (A) and with pauses (B). The time delays measured between proximal and distal injury sites were higher in simulations incorporating dyenin pauses, although the failure percentage of the system revealed no significant differences between these two model configurations (C). Three repetitions were performed for each model configuration.
(0.41 MB PDF)

Figure S7 Evaluating the effects of switching dynein velocities. In previous simulations, velocities are assigned at the beginning of each run, and a given molecule will travel with its initially assigned velocity throughout the entire simulation (A). Allowing 10% of the molecules to switch velocities once per 100 time steps during the simulations improved model performance (B). The effect of velocity switching, depicted in terms of failure percentage, is statistically significant for all tested sensitivity thresholds (C). Comparison of failure percentages between fixed and switching velocities is provided for two model configurations: a single slow signal configuration, and a multiple slow signals configuration (integrating 3 out of 6 slow signals). Panels (A) and (B) depict an analysis of total-distance/injury-displacement combinations for detector sensitivity threshold of 20% under fixed velocities simulations and switching velocities simulations, respectively.
(0.40 MB PDF)

## References

[1] Hanz S, Fainzilber M (2006). Retrograde signaling in injured nerve - the axon reaction revisited.. J Neurochem.

[2] Rossi F, Gianola S, Corvetti L (2007). Regulation of intrinsic neuronal properties for axon growth and regeneration.. Prog Neurobiol.

[3] Lu P, Tuszynski MH (2008). Growth factors and combinatorial therapies for CNS regeneration.. Exp Neurol.

[4] Benowitz L, Yin Y (2008). Rewiring the injured CNS: lessons from the optic nerve.. Exp Neurol.

[5] Mason MR, Lieberman AR, Anderson PN (2003). Corticospinal neurons up-regulate a range of growth-associated genes following intracortical, but not spinal, axotomy.. Eur J Neurosci.

[6] Zagrebelsky M, Buffo A, Skerra A, Schwab ME, Strata P (1998). Retrograde regulation of growth-associated gene expression in adult rat Purkinje cells by myelin-associated neurite growth inhibitory proteins.. J Neurosci.

[7] Buffo A, Carulli D, Rossi F, Strata P (2003). Extrinsic regulation of injury/growth-related gene expression in the inferior olive of the adult rat.. Eur J Neurosci.

[8] Kenney AM, Kocsis JD (1998). Peripheral axotomy induces long-term c-Jun amino-terminal kinase-1 activation and activator protein-1 binding activity by c-Jun and junD in adult rat dorsal root ganglia In vivo.. J Neurosci.

[9] Kenney AM, Kocsis JD (1997). Timing of c-jun protein induction in lumbar dorsal root ganglia after sciatic nerve transection varies with lesion distance.. Brain Res.

[10] Fernandes KJ, Fan DP, Tsui BJ, Cassar SL, Tetzlaff W (1999). Influence of the axotomy to cell body distance in rat rubrospinal and spinal motoneurons: differential regulation of GAP-43, tubulins, and neurofilament-M.. J Comp Neurol.

[11] Tsujino H, Kondo E, Fukuoka T, Dai Y, Tokunaga A (2000). Activating transcription factor 3 (ATF3) induction by axotomy in sensory and motoneurons: A novel neuronal marker of nerve injury.. Mol Cell Neurosci.

[12] Doster SK, Lozano AM, Aguayo AJ, Willard MB (1991). Expression of the growth-associated protein GAP-43 in adult rat retinal ganglion cells following axon injury.. Neuron.

[13] You S-W, So K-F, Yip HK (2000). Axonal Regeneration of Retinal Ganglion Cells Depending on the Distance of Axotomy in Adult Hamsters.. Invest Ophthalmol Vis Sci.

[14] Zottoli SJ, Hangen DH, Faber DS (1984). The axon reaction of the goldfish mauthner cell and factors that influence its morphological variability.. J Comp Neurol.

[15] Cancalon PF (1987). Survival and subsequent regeneration of olfactory neurons after a distal axonal lesion.. J Neurocytol.

[16] Cragg BG (1970). What is the signal for chromatolysis?. Brain Res.

[17] Lieberman AR (1971). The axon reaction: a review of the principal features of perikaryal responses to axon injury.. Int Rev Neurobiol.

[18] Kholodenko BN (2003). Four-dimensional organization of protein kinase signaling cascades: the roles of diffusion, endocytosis and molecular motors.. J Exp Biol.

[19] Markevich NI, Tsyganov MA, Hoek JB, Kholodenko BN (2006). Long-range signaling by phosphoprotein waves arising from bistability in protein kinase cascades.. Mol Syst Biol.

[20] Stelling J, Kholodenko BN (2009). Signaling cascades as cellular devices for spatial computations.. J Math Biol.

[21] Perlson E, Hanz S, Medzihradszky KF, Burlingame AL, Fainzilber M (2004). From snails to sciatic nerve: Retrograde injury signaling from axon to soma in lesioned neurons.. J Neurobiol.

[22] Abe N, Cavalli V (2008). Nerve injury signaling.. Curr Opin Neurobiol.

[23] Howe CL, Mobley WC (2004). Signaling endosome hypothesis: A cellular mechanism for long distance communication.. J Neurobiol.

[24] Ibanez CF (2007). Message in a bottle: long-range retrograde signaling in the nervous system.. Trends Cell Biol.

[25] Howe CL (2005). Modeling the signaling endosome hypothesis: why a drive to the nucleus is better than a (random) walk.. Theor Biol Med Model.

[26] Mandolesi G, Madeddu F, Bozzi Y, Maffei L, Ratto GM (2004). Acute physiological response of mammalian central neurons to axotomy: ionic regulation and electrical activity.. Faseb J.

[27] Mitchell CS, Lee RH (2009). A quantitative examination of the role of cargo-exerted forces in axonal transport.. J Theor Biol.

[28] Ross JL, Wallace K, Shuman H, Goldman YE, Holzbaur EL (2006). Processive bidirectional motion of dynein-dynactin complexes in vitro.. Nat Cell Biol.

[29] Deinhardt K, Salinas S, Verastegui C, Watson R, Worth D (2006). Rab5 and Rab7 control endocytic sorting along the axonal retrograde transport pathway.. Neuron.

[30] Hanz S, Perlson E, Willis D, Zheng JQ, Massarwa R (2003). Axoplasmic importins enable retrograde injury signaling in lesioned nerve.. Neuron.

[31] Perlson E, Hanz S, Ben-Yaakov K, Segal-Ruder Y, Seger R (2005). Vimentin-dependent spatial translocation of an activated MAP kinase in injured nerve.. Neuron.

[32] Yudin D, Hanz S, Yoo S, Iavnilovitch E, Willis D (2008). Localized regulation of axonal RanGTPase controls retrograde injury signaling in peripheral nerve.. Neuron.

[33] Loewy AD, Schader RE (1977). A quantitative study of retrograde neuronal changes in Clarke's column.. J Comp Neurol.

[34] Watson WE (1968). Observations on the nucleolar and total cell body nucleic acid of injured nerve cells.. J Physiol.

[35] Mallik R, Carter BC, Lex SA, King SJ, Gross SP (2004). Cytoplasmic dynein functions as a gear in response to load.. Nature.

[36] Gross SP, Welte MA, Block SM, Wieschaus EF (2000). Dynein-mediated cargo transport in vivo. A switch controls travel distance.. J Cell Biol.

[37] Rose CR, Blum R, Kafitz KW, Kovalchuk Y, Konnerth A (2004). From modulator to mediator: rapid effects of BDNF on ion channels.. Bioessays.

[38] Ha J, Lo KW, Myers KR, Carr TM, Humsi MK (2008). A neuron-specific cytoplasmic dynein isoform preferentially transports TrkB signaling endosomes.. J Cell Biol.

[39] Saha RN, Dudek SM (2008). Action potentials: to the nucleus and beyond.. Exp Biol Med (Maywood).

[40] Thompson KR, Otis KO, Chen DY, Zhao Y, O'Dell TJ (2004). Synapse to nucleus signaling during long-term synaptic plasticity; a role for the classical active nuclear import pathway.. Neuron.

[41] Dieterich DC, Karpova A, Mikhaylova M, Zdobnova I, Konig I (2008). Caldendrin-Jacob: a protein liaison that couples NMDA receptor signalling to the nucleus.. PLoS Biol.

[42] Ambros V (1999). Cell cycle-dependent sequencing of cell fate decisions in Caenorhabditis elegans vulva precursor cells.. Development.

[43] Lenschow DJ, Walunas TL, Bluestone JA (1996). CD28/B7 system of T cell costimulation.. Annu Rev Immunol.

[44] Nelder JA, Mead R (1965). A Simplex Method for Function Minimization.. The Computer Journal.

[45] Lagarias JC, Reeds JA, Wright MH, Wright PE (1998). Convergence Properties of the Nelder-Mead Simplex Method in Low Dimensions.. SIAM Journal of Optimization.

