# Supplementary figures and images for: Can Molecular Motors Drive Distance Measurements in Injured Neurons?
Source: PLoS Comput Biol. 2009 Aug 21;5(8):e1000477. doi: 10.1371/journal.pcbi.1000477 (PMC2718615; doi:10.1371/journal.pcbi.1000477)

Figure S1

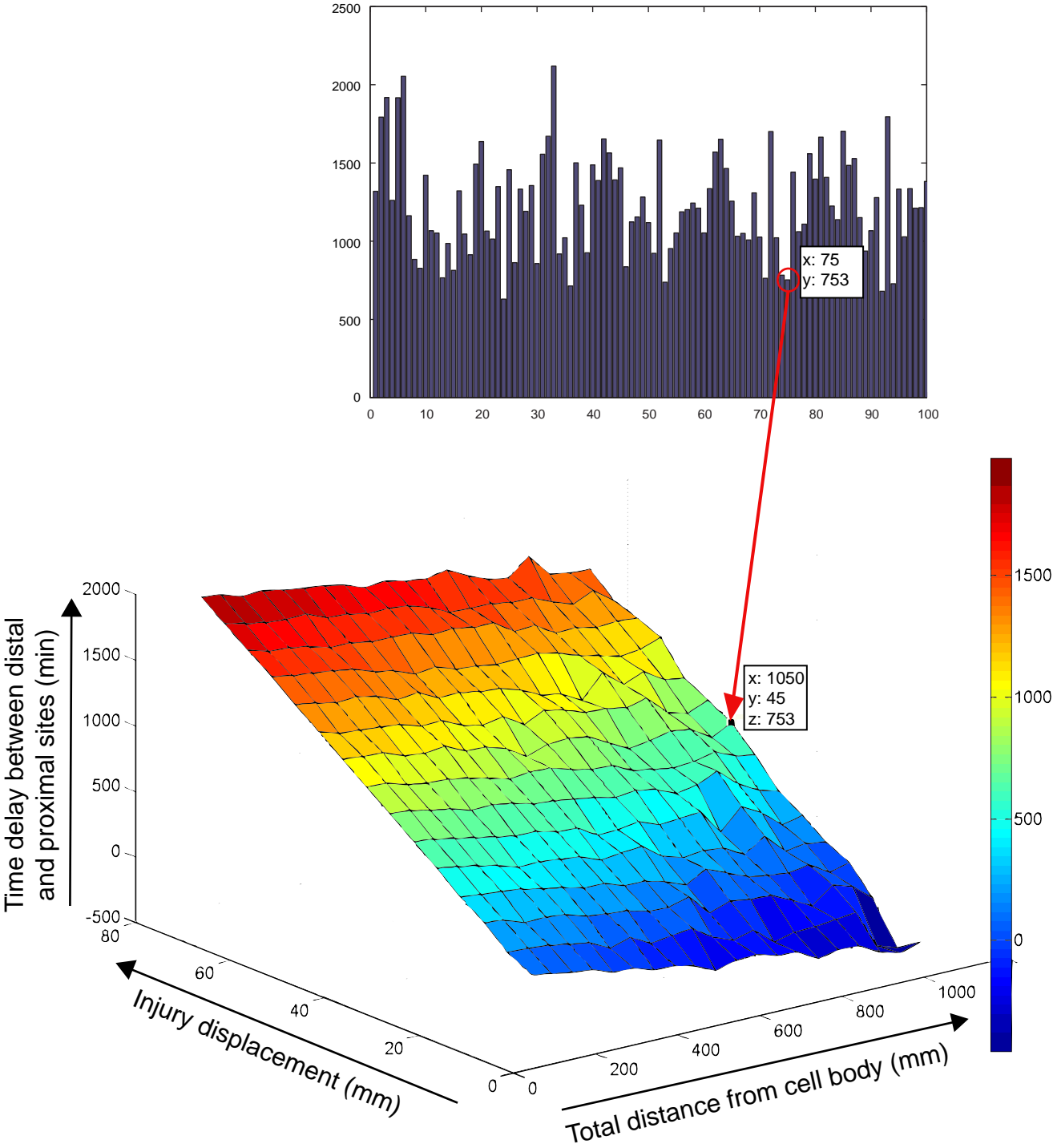

Supplement: Figure S1 — Systematic analysis of total distance and injury displacement combinations. The X axis represents total distance from cell body (mm), the Y axis represents injury displacement (mm), and the Z axis represents the minimal (i.e. worst case) Δt2−Δt1 time-difference value (in minutes) out of 100 simulation repeats obtained for each X–Y combination. The figure shows results for a 20% sensitivity threshold in a multiple-signal based model. (0.34 MB PDF) [file pcbi.1000477.s001.pdf]

Figure S2

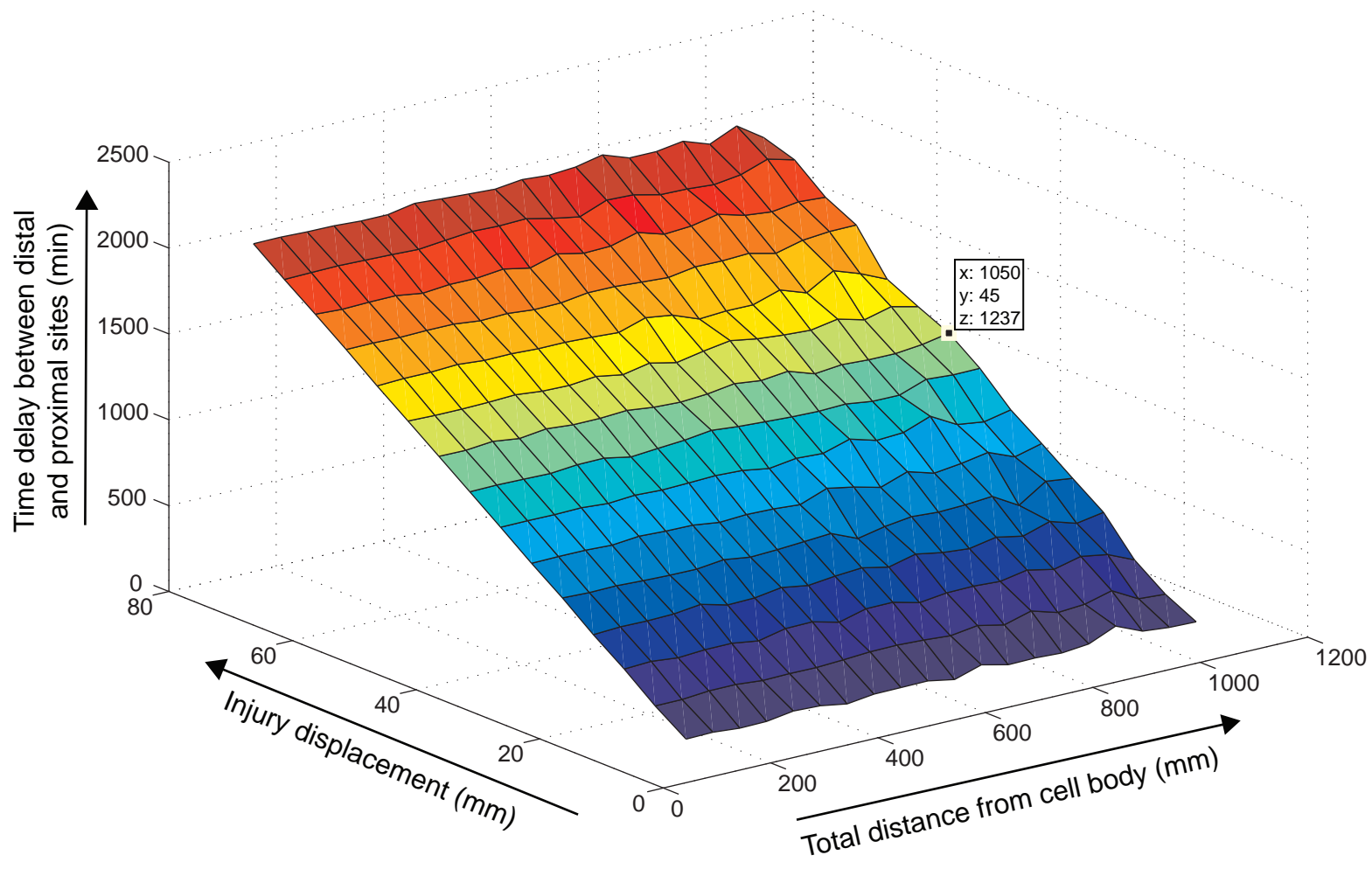

Supplement: Figure S2 — An “ideal” graph. Each data point represents the mean Δt2−Δt1 time-difference value (in minutes) of 100 simulation repeats obtained for each X–Y combination. The figure shows data for a 20% sensitivity threshold in a multiple-signal based model. (0.15 MB PDF) [file pcbi.1000477.s002.pdf]

**Figure S3**

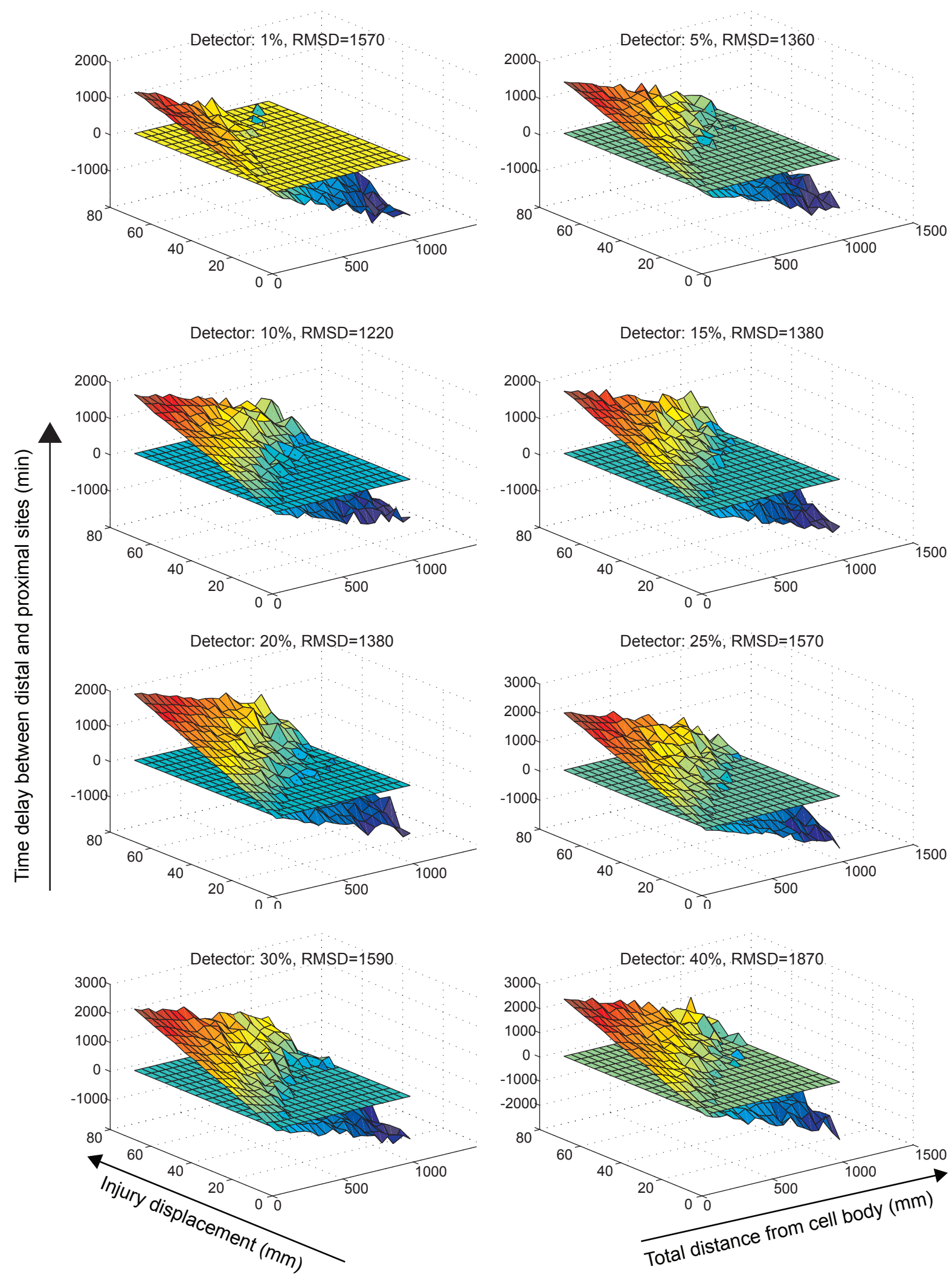

Figure S3

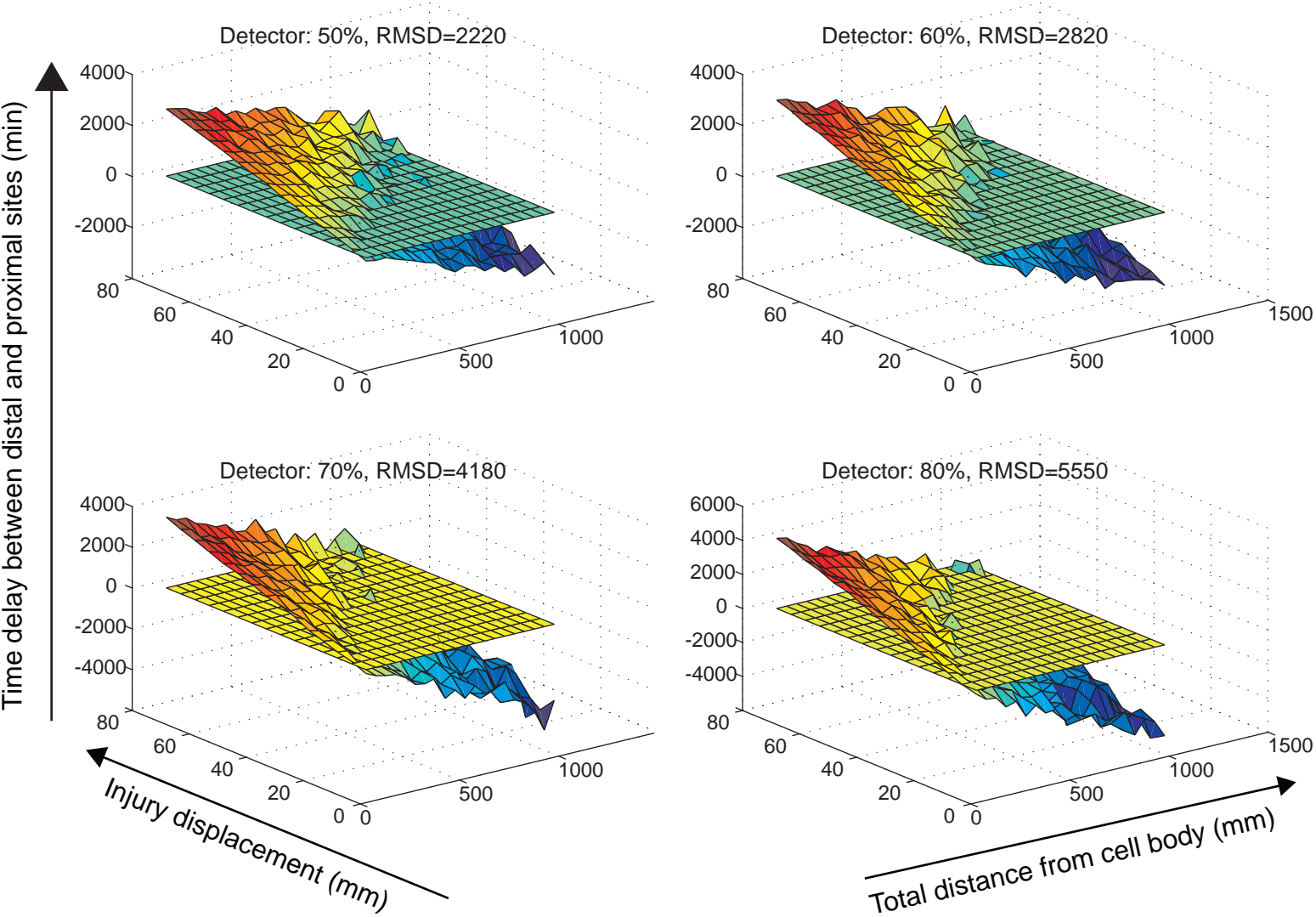

Supplement: Figure S3 — The influence of detector sensitivity threshold on a two-signals model performance. Simulations were run for a wide range of detector sensitivity thresholds, revealing an optimal performance at the range of 10%–20%. RMSD values are depicted for each model configuration. (1.27 MB PDF) [file pcbi.1000477.s003.pdf]

**Figure S4**

**A**

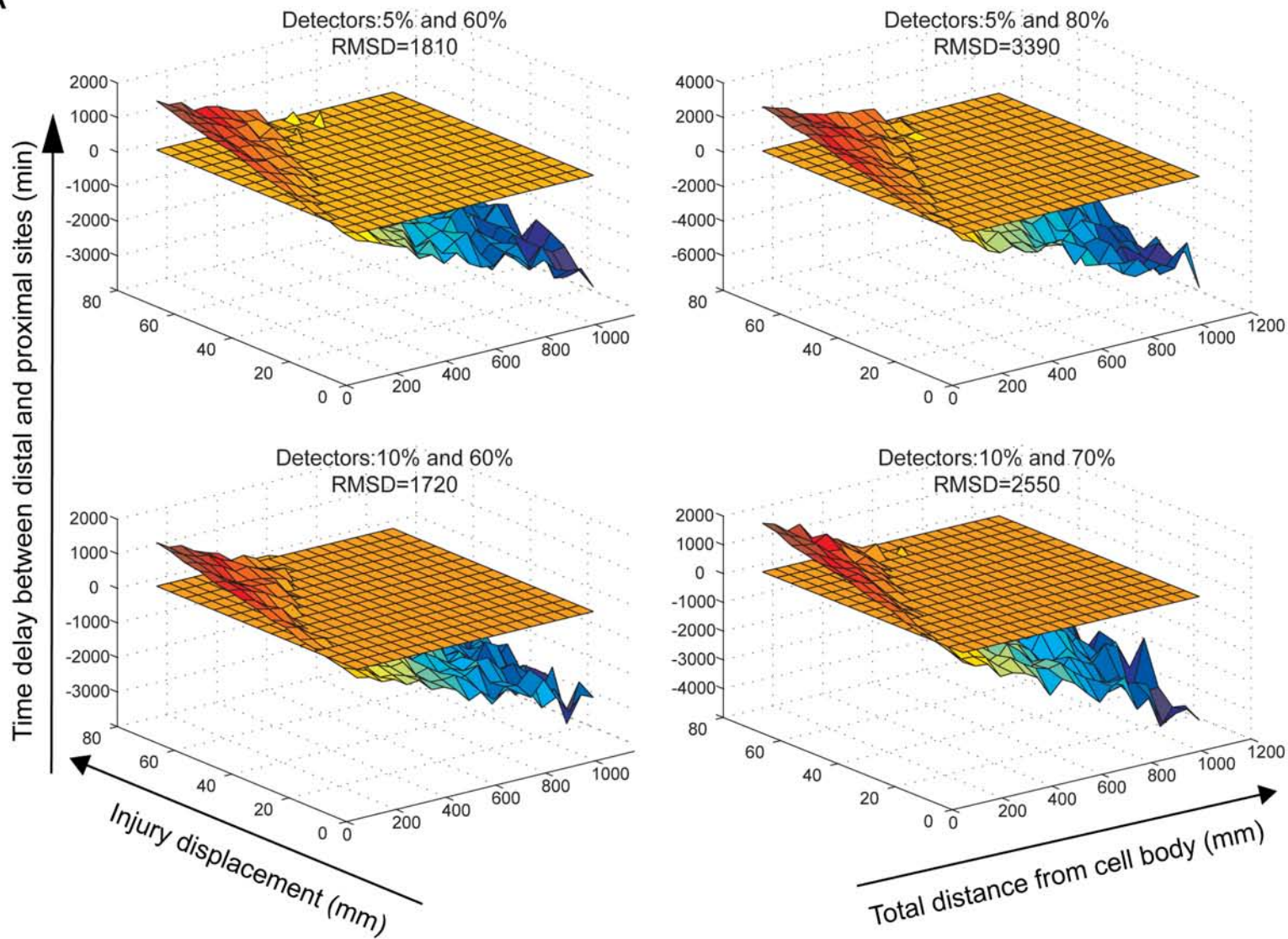

**B**

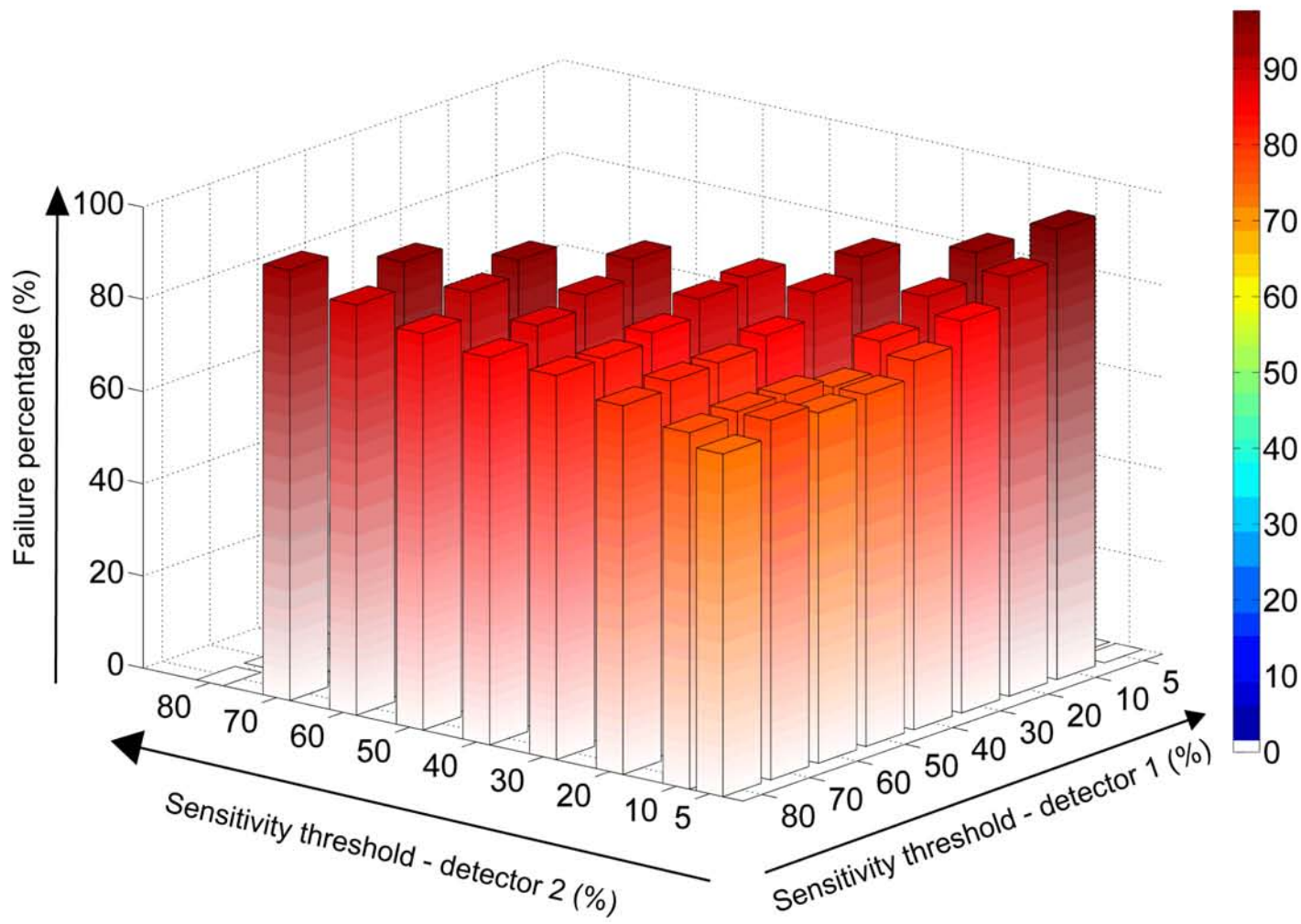

Supplement: Figure S4 — The influence of detector sensitivity threshold on a two-detectors model performance. A wide range of detector sensitivity combinations was examined, but failed to exceed a success rate of 35% in distinguishing between proximal and distal injuries. (A) Depicted examples include the following detector sensitivity threshold combinations: 5% and 60%, 5% and 80%, 10% and 70%, and 10% and 60%. (B) Failure percentage in various combinations of two detectors. The X axis (left) represents the sensitivity threshold of the more sensitive detector (Detector 1), whereas the Y axis represents the sensitivity threshold of the less sensitive detector (Detector 2). The lowest failure percentage was received for the 5%-and-80% configuration. Configurations in which the two detectors had relatively similar sensitivity threshold (back diagonal) gave the poorest performance. (1.51 MB PDF) [file pcbi.1000477.s004.pdf]

Figure S5

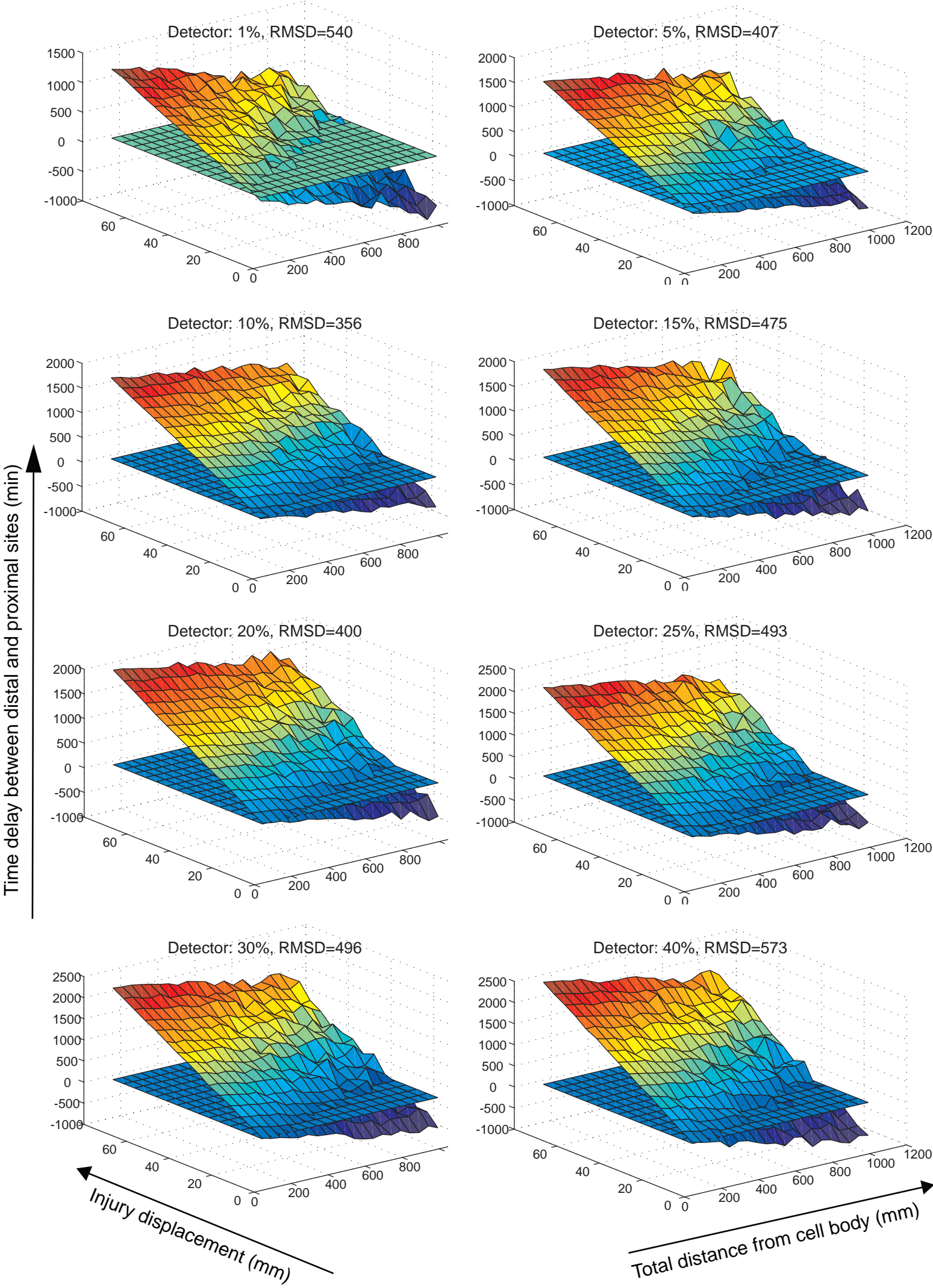

Figure S5

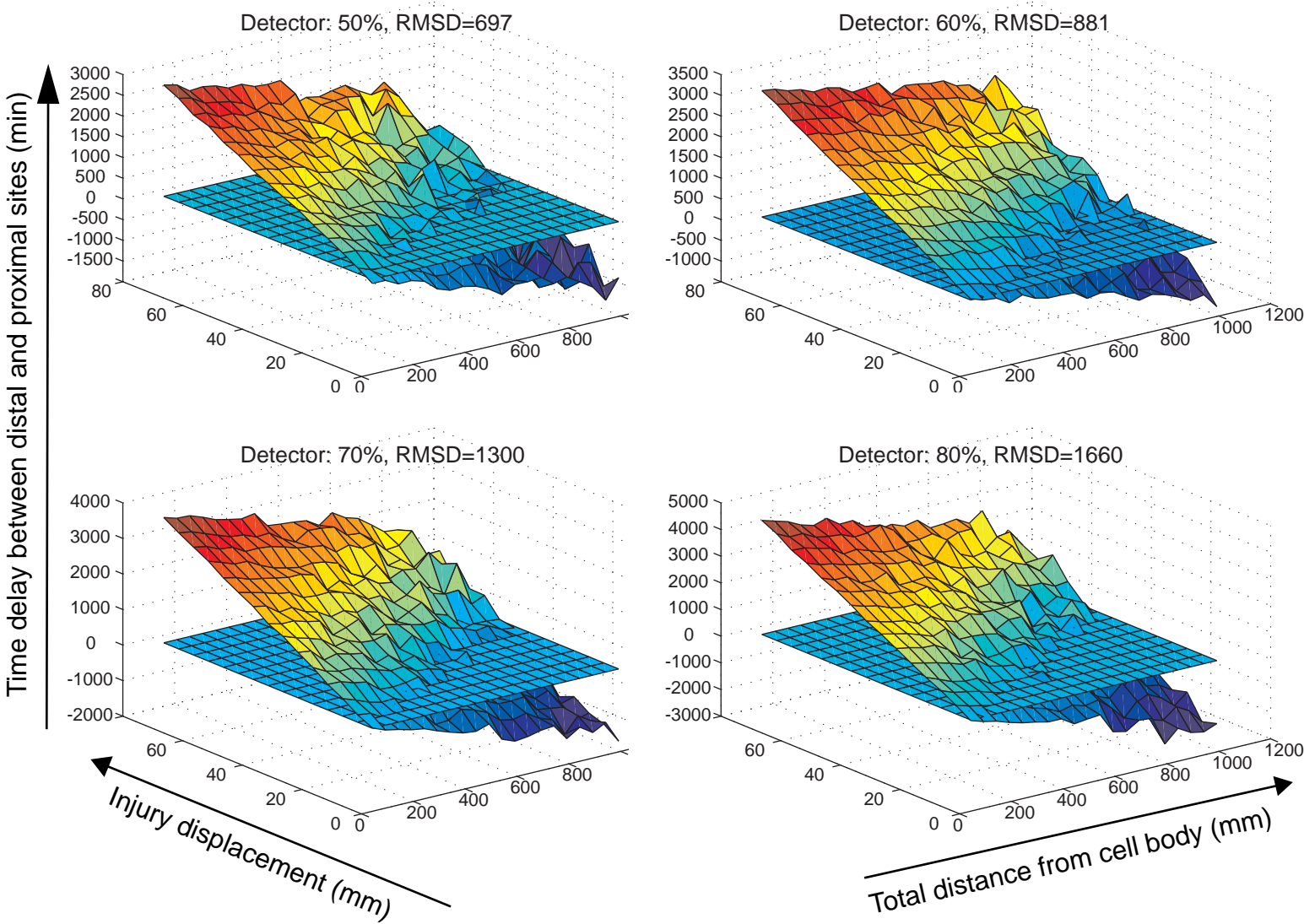

Supplement: Figure S5 — Influence of detector sensitivity on performance of the multiple signals model. Simulations were run for a wide range of detector sensitivity thresholds, revealing an optimal performance in the range of 10%–40%. RMSD values are depicted for each model configuration. Note that not only did model extension improve the performance of a given detector sensitivity threshold, but moreover the worst performing configuration of the multiple signals system was still better than the best performance of the single slow signal system. In addition, the range of “optimal detectors” in a multiple-signals system is wider - 10–40% compared to 10–20% for the original system (see Fig. S3). (1.44 MB PDF) [file pcbi.1000477.s005.pdf]

**Figure S6**

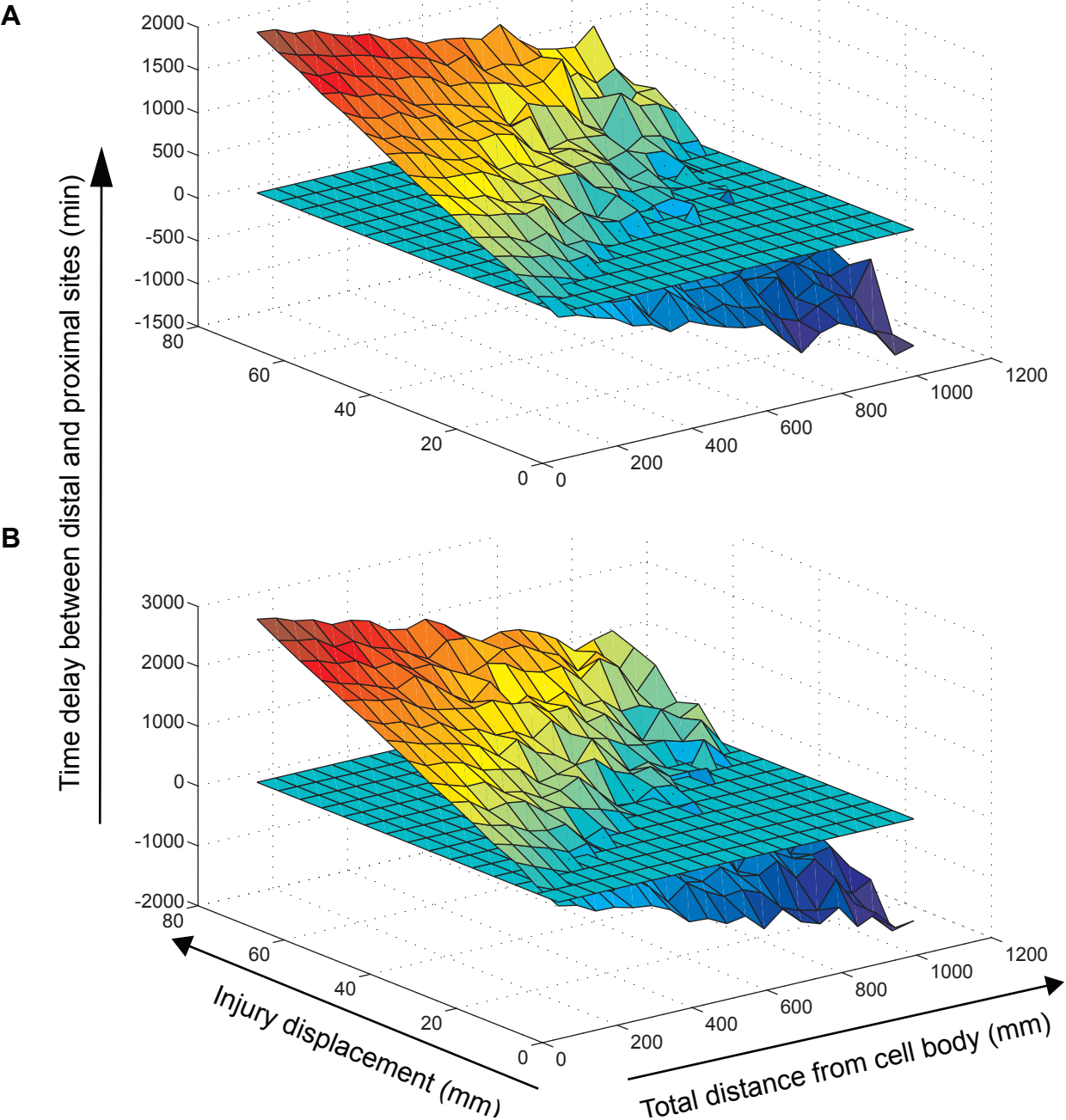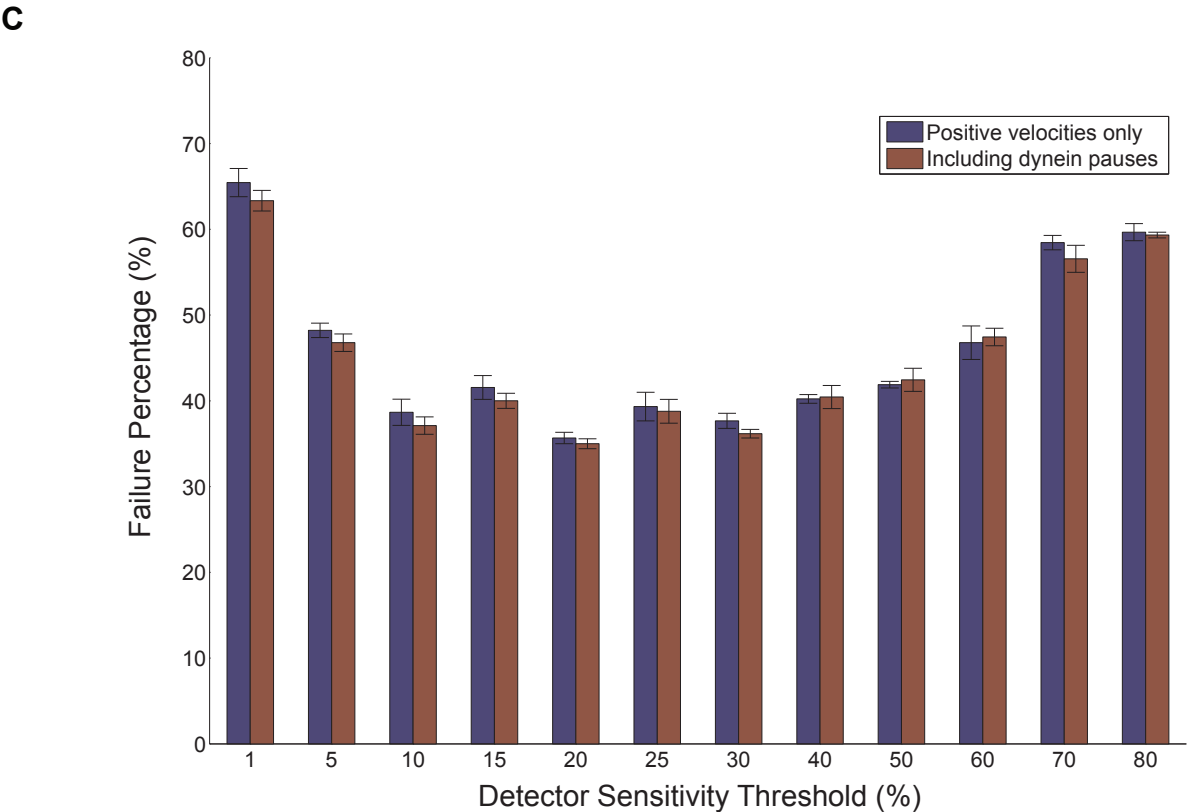

Supplement: Figure S6 — Evaluation of the effect of dynein pauses in the two-signals model. The velocity distributions depicted in most of our analyses refer only to positive dynein velocities, although it has been shown that dynein movement may also include pauses (velocity = 0), as well as limited movements in the opposite direction (i.e., negative velocity). We therefore ran a set of simulations in which 30% of the particles were randomly assigned to pause at any given time step. Paused particles resumed movement at their originally assigned velocity at the subsequent time step. Panels A and B depict the results of simulations for a model configuration with one slow signal and a detector sensitivity of 20%, without pauses (A) and with pauses (B). The time delays measured between proximal and distal injury sites were higher in simulations incorporating dyenin pauses, although the failure percentage of the system revealed no significant differences between these two model configurations (C). Three repetitions were performed for each model configuration. (0.41 MB PDF) [file pcbi.1000477.s006.pdf]

Figure S7

A

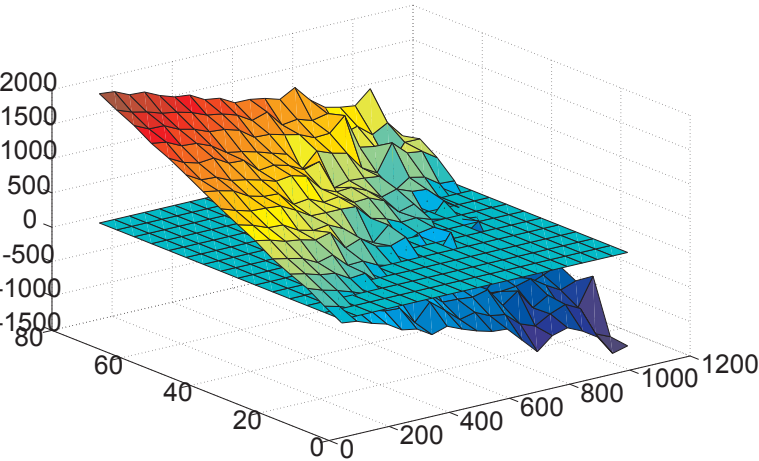

B

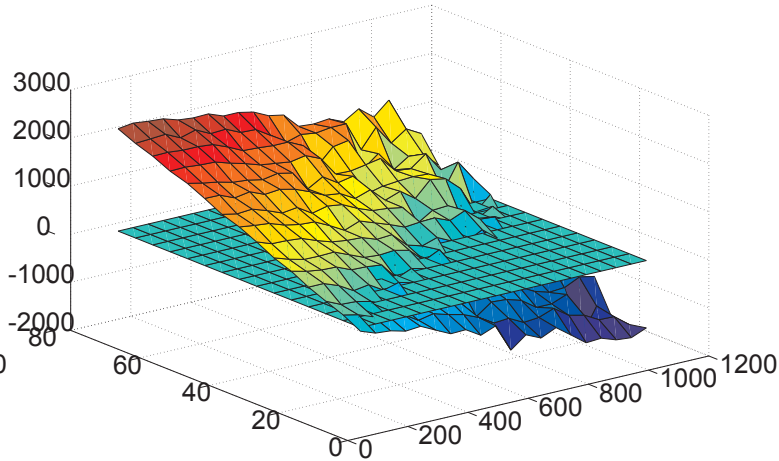

C

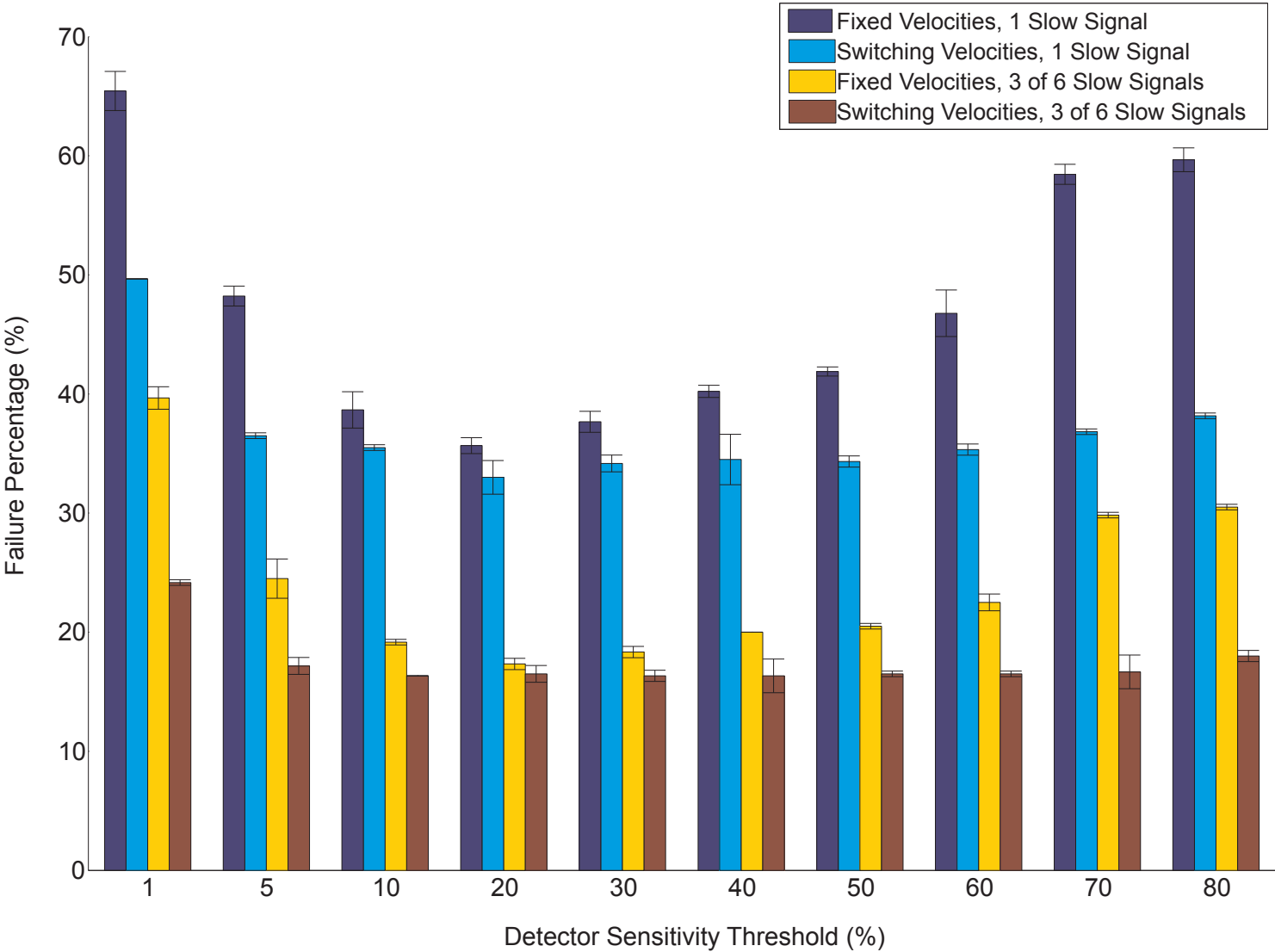

Supplement: Figure S7 — Evaluating the effects of switching dynein velocities. In previous simulations, velocities are assigned at the beginning of each run, and a given molecule will travel with its initially assigned velocity throughout the entire simulation (A). Allowing 10% of the molecules to switch velocities once per 100 time steps during the simulations improved model performance (B). The effect of velocity switching, depicted in terms of failure percentage, is statistically significant for all tested sensitivity thresholds (C). Comparison of failure percentages between fixed and switching velocities is provided for two model configurations: a single slow signal configuration, and a multiple slow signals configuration (integrating 3 out of 6 slow signals). Panels (A) and (B) depict an analysis of total-distance/injury-displacement combinations for detector sensitivity threshold of 20% under fixed velocities simulations and switching velocities simulations, respectively. (0.40 MB PDF) [file pcbi.1000477.s007.pdf]
